# Interleukin-11 signaling promotes cellular reprogramming and limits fibrotic scarring during tissue regeneration

**DOI:** 10.1126/sciadv.abg6497

**Published:** 2021-09-08

**Authors:** Srinivas Allanki, Boris Strilic, Lilly Scheinberger, Yeszamin L. Onderwater, Alora Marks, Stefan Günther, Jens Preussner, Khrievono Kikhi, Mario Looso, Didier Y. R. Stainier, Sven Reischauer

**Affiliations:** 1 Department of Developmental Genetics, Max Planck Institute for Heart and Lung Research, 61231 Bad Nauheim, Germany.; 2 German Centre for Cardiovascular Research (DZHK), Partner Site Rhine-Main, 60596 Frankfurt am Main, Germany.; 3 Medical Clinic I (Cardiology/Angiology) and Campus Kerckhoff, Justus-Liebig-University Giessen, 35392 Giessen, Germany.; 4 Department of Pharmacology, Max Planck Institute for Heart and Lung Research, 61231 Bad Nauheim, Germany.; 5 Bioinformatics and Deep Sequencing Platform, Max Planck Institute for Heart and Lung Research, 61231 Bad Nauheim, Germany.; 6 Bioinformatics Core Unit (BCU), Max Planck Institute for Heart and Lung Research, 61231 Bad Nauheim, Germany.; 7 Flow Cytometry Service Group, Max Planck Institute for Heart and Lung Research, 61231 Bad Nauheim, Germany.; 8 Cardio-Pulmonary Institute, Frankfurt, Germany.

## Abstract

Damage-induced fibrotic scarring limits tissue regeneration in mammals and is a leading cause of morbidity. In contrast, species like zebrafish can regenerate damaged tissues without excessive fibrosis. However, whether specific signaling pathways can both limit fibrosis and promote regeneration is unclear. Here, we show that interleukin-11 (Il-11)/Stat3 signaling has such a dual function. Zebrafish lacking Il-11 receptor function display severely compromised heart, fin, and scale regeneration. Deep phenotyping and transcriptional analysis of adult hearts and fins show that Il-11 signaling drives cellular reprogramming to orchestrate global and tissue-specific regenerative programs and broadly antagonizes hallmarks of adult mammalian scarring. Mechanistically, our data indicate that IL-11 signaling in endothelial cells antagonizes profibrotic transforming growth factor–β signaling and endothelial-to-mesenchymal transition, limiting scarring and promoting cardiomyocyte repopulation, after injury. Overall, our findings position damage-induced Il-11/Stat3 signaling in a key role limiting fibrosis and promoting regeneration, revealing novel targets for regenerative therapies.

## Teaser

The cytokine interleukin-11 promotes regeneration and limits scar formation in multiple zebrafish tissues after injury.

## INTRODUCTION

**Fig. 1.**
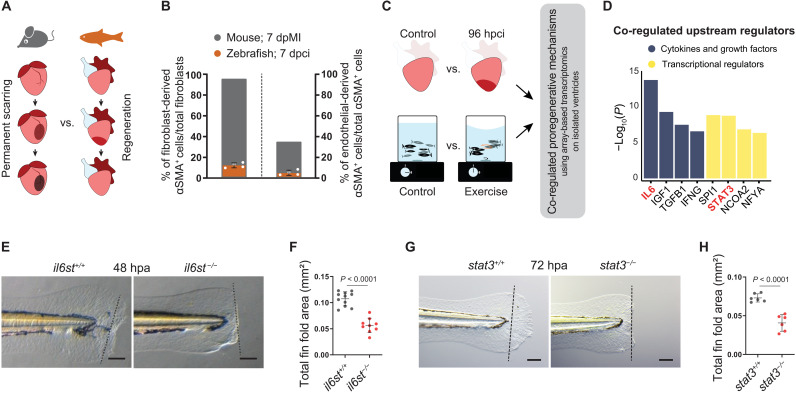
Il-6 cytokine family–mediated Stat3 signaling is proregenerative. (**A**) Illustration of scarring in an adult mammalian heart in contrast to regeneration in an adult zebrafish heart. (**B**) Quantification of epicardial- and endothelial-derived αSMA^+^ cells after MI in mouse [fibroblasts (*3*); endothelial cells (*4*)] and 7 dpci in zebrafish [fibroblasts, *Tg*(*tcf21:CreER*), *n* = 4; endothelial cells, *Tg*(*kdrl:Cre*), *n* = 4]. (**C**) Schematics of the comparative transcriptional profiling. (**D**) Results from upstream regulator prediction analysis using Ingenuity Pathway Analysis. (**E** to **H**) Bright-field images of larval fin fold regeneration (E and G) [amputated at 48 to 60 hours post fertilization (hpf)] and their corresponding quantification of the fin fold area [(F) (*il6st^sa1462^*, wt siblings, *n* = 11; mut, *n* = 9, 48 hpa; (H) *stat3^stl27^*, wt siblings, *n* = 6; mut, *n* = 6, 72 hpa]. Data represent means ± SD (B, F, and H). Student’s *t* tests (F and H). *n* = larvae (F and H). Black dashed lines demarcate the amputation plane (E and G). Scale bars, 100 μm (E and G).

Severe organ damage culminates in tissue regeneration or the formation of a permanent and functionally inert scar (*1*). In particular, adult mammals respond to tissue damage with fibrotic scarring, which is characterized by the activation of fibrotic gene programs, myofibroblast differentiation, and the deposition of a stiff, collagen-rich extracellular matrix (ECM) (Fig. 1A) (*2*). After cardiac injury, α–smooth muscle actin (αSMA)^+^ myofibroblasts arise from cardiac fibroblasts and endothelial cells and are the principal contributors to scar-like ECM remodeling (*3*–*5*). Myofibroblasts can further differentiate into more specialized matrifibrocytes to support the mature scar (*3*). Notably, eliminating myofibroblasts in mice indeed limits fibrotic remodeling and promotes functional recovery after injury (*6*–*8*).

In contrast to mammals, some vertebrates, including zebrafish, rebuild damaged organs and appendages throughout life, with only limited and transient deposition of a matrix (Fig. 1A) (*9*–*12*). Regenerative species, after tissue damage, undergo cellular reprogramming by switching from homeostatic gene expression to a regeneration-specific gene program to activate vital cellular processes, including dedifferentiation, proliferation, and migration, allowing regeneration (*13*–*17*). Although scar formation clearly correlates negatively with regeneration, the existence of specific upstream mechanisms that both promote cellular reprogramming and limit scarring is unclear. In this study, through genetic loss-of-function approaches, lineage tracing, the examination of regeneration in various tissues and developmental stages, and combinatorial transcriptome profiling, we show that interleukin-11 (Il-11)/signal transducer and activator of transcription 3 (Stat3) signaling plays two roles: (i) It promotes cellular reprogramming by orchestrating regenerative programs, and (ii) it limits injury-induced mammalian-like scarring.

## RESULTS

### Il-6 cytokine family–mediated Stat3 signaling is proregenerative

First, to compare the scarring response in adult zebrafish and mouse hearts, we analyzed myofibroblast differentiation after cardiac cryoinjury in zebrafish. While ~95% of cardiac fibroblasts have been reported to differentiate into myofibroblasts in adult mice after myocardial infarction (MI) (*3*), we found, using lineage tracing, that in zebrafish, this response was strongly limited (12.27 ± 2.40%) (Fig. 1B and fig. S1A). Similarly, we found in zebrafish only a very minor endothelial contribution to myofibroblasts after injury [mouse, ~35% (*4*); zebrafish, 4.03 ± 2.45%] (Fig. 1B and fig. S1B). On the basis of these data, we hypothesized that in zebrafish, inherent mechanisms limit the myofibroblast-mediated scarring response after tissue damage, allowing efficient regeneration. To identify these mechanisms, we first profiled the transcriptome of regenerating zebrafish ventricles (Fig. 1C). However, cardiac injury alters a substantial proportion of the transcriptome [16% of the probe set; fold change (FC) ≥ ±2] in agreement with previous reports (*16*, *18*), rendering candidate identification difficult. To narrow down the number of candidate genes, we established a second dataset of transcriptional changes by modeling in zebrafish moderate physical activity (Fig. 1C), a well-known approach to ameliorate tissue remodeling in human cardiomyopathies (*19*). Combining comparative pathway and upstream regulator prediction analyses on 180 co-regulated genes (table S1), we identified Il-6 cytokine family–mediated Janus kinase (Jak)–Stat3 signaling as a promising candidate pathway (Fig. 1D, fig. S2A, and table S2).

The Il-6 family in zebrafish consists of seven evolutionarily conserved cytokines, each binding to a specific receptor (fig. S2, B to D). These ligand-receptor complexes further heterodimerize with the common co-receptor Interleukin-6 signal transducer (Il6st; also known as Gp130). The intracellular domain of Il6st is responsible for further signal transduction, mostly through the canonical Jak-Stat3 pathway, as well as through the noncanonical mitogen-activated protein kinase kinase (Mek)/extracellular signal–regulated kinase (Erk) and phosphatidylinositol 3‑kinase (Pi3k)/protein kinase B (Akt) pathways. To investigate the potential role of canonical Il-6 family signaling during regeneration and scarring, we used the loss-of-function alleles, *il6st^sa1462^* and *stat3^stl27^*. As observed in the respective mouse mutants (*20*, *21*), *il6st* and *stat3* mutant zebrafish do not survive to adulthood, with only a few escapers alive at 8 weeks post fertilization (*22*). These animals display severe skeletal abnormalities and thus are not suitable for adult regeneration studies (fig. S2, E and F). However, the mutant larvae are morphologically indistinguishable from their wild-type siblings, allowing the investigation of fin fold regeneration. In line with previous observations (*23*), we found that *stat3* mutant larvae exhibit a severely compromised regenerative potential after fin fold amputation (Fig. 1, G and H), as do *il6st* mutants (Fig. 1, E and F), suggesting a requirement for canonical Il6st/Stat3 signaling during regeneration.

### Il-11 signaling is indispensable for scar-free regeneration in diverse tissues

**Fig. 2.**
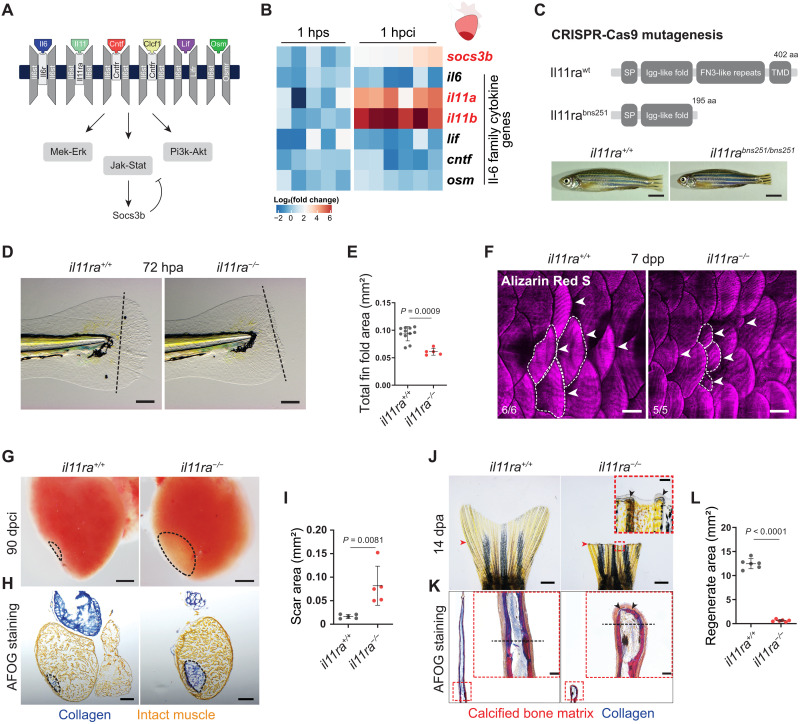
Il-11 signaling is essential for scar-free regeneration. (**A**) Illustration of zebrafish Il-6 family cytokines and receptors and the downstream signaling pathways. (**B**) Heatmap showing RT-qPCR analysis of Il-6 family cytokine gene mRNA levels at 1 hpci (*n* = 6) compared with 1 hour post sham (hps; *n* = 5). (**C**) Illustration of wild-type and predicted mutant proteins and gross morphology of adult zebrafish siblings. (**D** and **E**) Bright-field images of larval fin fold regeneration (D) (wt siblings, *n* = 11; mut, *n* = 5; 72 hpa) and their corresponding quantification of the total fin fold area (E). (**F**) Wholemount images of Alizarin Red S stained regenerating adult scales [wt siblings, *n* = 6; mut, *n* = 5; 7 days post plucking (dpp)]. (**G** to **I**) Wholemount images of cardiac ventricles (G) (wt siblings, *n* = 5; mut, *n* = 5; 90 dpci), Acid Fuchsin Orange G (AFOG) staining on cryosections (H), and quantification of the scar area (I). (**J** to **L**) Wholemount images of caudal fins (J) [wt, *n* = 6; mut, *n* = 6, 14 days post amputation (dpa)], AFOG staining on longitudinal cryosections (K), and quantification of the regenerate area (L). SP, signal peptide; TMD, transmembrane domain; aa, amino acids (C). Data represent means ± SD (E, I, and L). Student’s *t* tests (E, I, and L). *n*, ventricles (B and G); *n*, larvae (D); *n*, adult zebrafish (F); *n*, caudal fins (J). Black dashed lines demarcate the injured area (G and H) and amputation plane (D and K); white dashed lines demarcate and white arrowheads point to regenerating scales (F); black arrowheads point to fused hemirays [insets in (J) and (K)]; red arrowheads point to the amputation plane (J). Ct values are listed in table S5. Scale bars, 5 mm (C), 100 μm (D), 500 μm (F), 200 μm (G and H), 1 mm (J), and 50 μm (K).

To identify the specific ligand modulating Il6st/Stat3 activity during regeneration, we assessed the expression levels of the seven Il-6 family cytokine genes after injury (Fig. 2A). We found that both the paralogous genes encoding Il-11 (*il11a* and *il11b*) were the most significantly induced and most highly expressed after tissue damage in the cardiac ventricle [1 hour post cryoinjury (hpci)] and caudal fins [1 hour post amputation (hpa)] (Fig. 2B and fig. S2G). Notably, published transcriptome data from various regenerating tissues in zebrafish (*24*), African killifish (*13*), lungfish (*25*), *Xenopus* (*26*), and axolotl (*15*) show an evolutionarily conserved and injury-responsive Il-11 induction. Spatial expression pattern analysis using RNA in situ hybridization in the cardiac ventricle at 24 hpci showed that both *il11a* and *il11b* are induced by various cell types but largely restricted to border zone endothelial cells (fig. S2H). Reverse transcription quantitative polymerase chain reaction (RT-qPCR) on sorted cells confirmed that endothelial cells express higher levels of *il11a* and *il11b* mRNA compared with nonendothelial cells at 96 hpci (fig. S2I). Similar analysis in the adult caudal fin showed that *il11a* is highly enriched at the amputation plane at 24 hpa (fig. S2J). Furthermore, we observed a robust sixfold induction of *socs3b*, a direct Stat3 target gene (Fig. 2B and fig. S2G), suggesting an early activation of Jak-Stat3 signaling in both the heart and caudal fin after injury. Previous studies have suggested a role for Il-6 family cytokine signaling in cardiomyocyte (CM) dedifferentiation and proliferation after cardiac injury (*24*, *27*, *28*). Hence, we inactivated Il-11 signaling in zebrafish by generating loss-of-function alleles for the specific receptor, the Il-11 receptor alpha–encoding gene *il11ra*, and the ligand-encoding genes *il11a* and *il11b* (fig. S2, B to D), using the CRISPR-Cas9 technology. This approach resulted in the isolation of three alleles, *il11ra^bns251^*, *il11a^bns311^*, and *il11b^bns312^* (Fig. 2C and fig. S3, A and D). *il11ra* and *il11a* mutant larvae display severe regeneration defects after fin fold amputation (Fig. 2, D and E, and fig. S3, B and C), similar to those observed in *il6st* and *stat3* mutants, while *il11b* mutants do not (fig. S3, E and F). Moreover, *il11ra* mutant zebrafish, unlike *il6st* and *stat3* mutants, and similar to *Il11ra1* mutant mice (*29*), survive to adulthood without overt developmental defects, suggesting that Il-11 signaling is largely dispensable for normal development, allowing the investigation of regeneration in adult stages (Fig. 2C). Strikingly, we observed that *il11ra* mutants display a severe loss of regenerative potential in diverse tissues and at various developmental stages, including the larval fin fold (Fig. 2D), adult heart (Fig. 2, G to I), adult caudal fin (Fig. 2, J to L), and adult scales (Fig. 2F). Specifically, adult *il11ra* mutants develop a permanent and collagen-rich scar lasting at least until 90 days post cardiac cryoinjury (dpci), a time point at which heart regeneration in wild types is nearly complete (Fig. 2, G to I, and fig. S4A). Similarly, both adult caudal fin amputation (Fig. 2J) and bone crush injury (fig. S4B) fail to induce regeneration in *il11ra* mutants. In particular, fin amputation in *il11ra* mutants leads to a complete lack of fin outgrowth and permanent hemiray fusion (Fig. 2, J to L, and fig. S4C), while *il11a* mutants (fig. S4D) and *il11ra* and *stat3* double heterozygotes (*il11ra^+/−^;stat3^+/−^*) display compromised regeneration (fig. S5). To test whether Il-11 signaling is needed in an injury-specific manner, we generated a transgenic line allowing a conditional reexpression of *il11ra* under the control of a heat shock promoter (*hsp70l*) in the *il11ra* mutant background (fig. S4E). Injury-specific reexpression of *il11ra* substantially rescued the *il11ra* mutant fin regeneration defects after amputation (fig. S4, E to G). Together, the genetic loss-of-function analysis of the ligands (Il-11a and Il-11b), the receptors (Il11ra and Il6st), and the Stat3 transcription factor shows that Il-11a/Il11ra/Il6st/Stat3 signaling is indispensable for regeneration in zebrafish.

### Il-11 signaling orchestrates cellular reprogramming and repopulation after injury

**Fig. 3.**
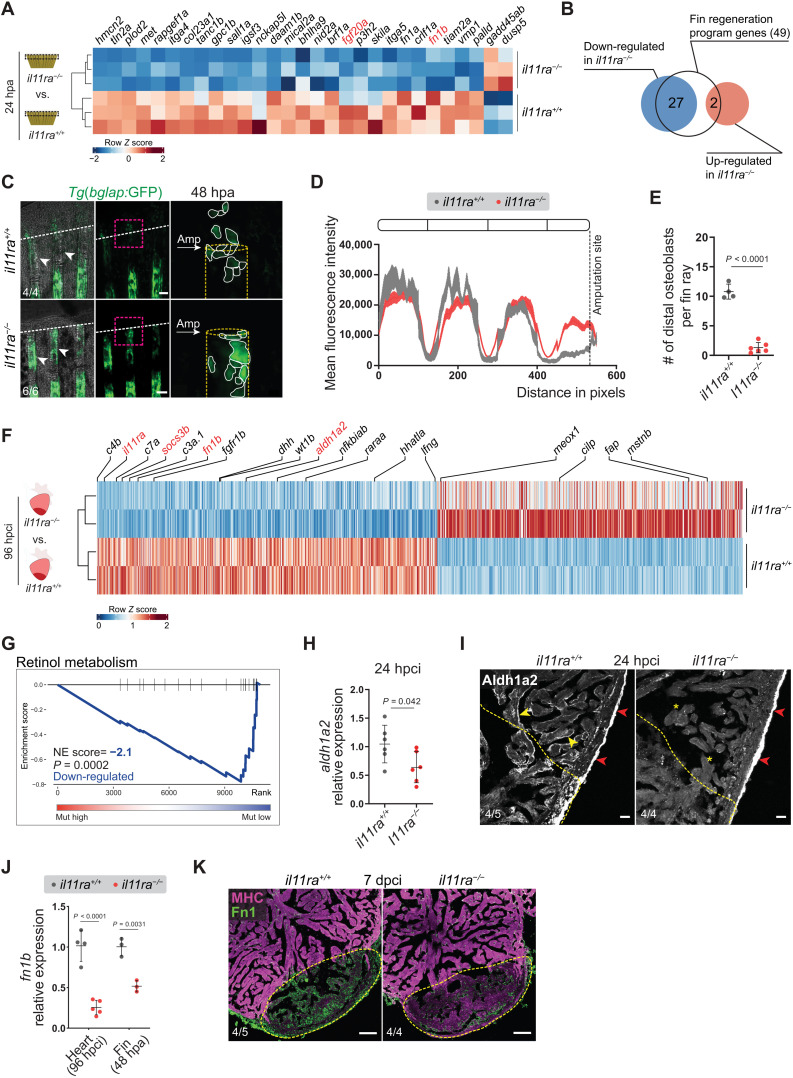
Il-11 signaling is required for the activation of global and tissue-specific regeneration gene programs. (**A** and **B**) Differential expression of fin regeneration genes (*13*) in *il11ra^−/−^* versus wild-type sibling caudal fin transcriptomic analysis, 24 hpa. (**C** to **E**) Confocal images of *Tg*(*bglap:*GFP) expression in wholemount caudal fins (C) (wt siblings, *n* = 4; mut, *n* = 6; 48 hpa), quantification of *Tg*(*bglap:*GFP) fluorescence intensity (D), and the number of distal osteoblasts (E). (**F**) Differential expression of known regulators of zebrafish cardiac regeneration (*35*) in *il11ra^−/−^* versus wild-type sibling ventricle transcriptomic analysis, 96 hpci. (**G**) GSE analysis plot of KEGG Retinol metabolism from *il11ra^−/−^* versus wild-type sibling ventricle transcriptomic analysis, 96 hpci. NE score, normalized enrichment score. (**H** and **I**) RT-qPCR analysis of *aldh1a2* mRNA levels (H) (wt siblings, *n* = 6; mut, *n* = 6; 24 hpci) and immunostaining for Aldh1a2 expression on cardiac ventricle cryosections (I) (wt siblings, *n* = 5; mut, *n* = 4; 24 hpci). (**J** and **K**) RT-qPCR analysis of *fn1b* mRNA levels on dissected injured areas from cardiac ventricles (J) (wt siblings, *n* = 4; mut, *n* = 5; 96 hpci) and caudal fins (J) (wt siblings, *n* = 3; mut, *n* = 3; 48 hpa) and immunostaining on cryosections (K) (wt siblings, *n* = 5; mut, *n* = 4; 7 dpci) for Fibronectin1 (green) and myosin heavy chain (MHC; magenta) expression. Data represent means ± SD (E, H, and J); means ± SEM (D). Student’s *t* tests (E, H, and J). *n*, ventricles [(H), (I), (J) heart, and (K)]; *n*, caudal fins [(C) and (J) fin]. White arrows point to and white dashed lines demarcate the amputation plane (C); white arrowheads point to *Tg*(*bglap:*GFP) expression (C); yellow dashed lines demarcate the injured area (I and K) and bone rays (C); yellow arrowheads and asterisks indicate endocardial Aldh1a2 expression (I); red arrowheads point to epicardial Aldh1a2 expression (I). Ct values are listed in table S5. Scale bars, 100 μm (C and K) and 20 μm (I).

To investigate the role of Il-11 signaling during regeneration, we performed RNA sequencing (RNA-seq). We profiled the transcriptomes of whole cardiac ventricles at 96 hpci and of caudal fin tissue at 24 hpa from *il11ra* mutants and wild-type siblings (Fig. 3, A and F). Realizing that the nonregenerative *il11ra* mutant fins display an impaired activation of prominent fin regeneration genes, including *devoid of blastema* (*dob*/*fgf20a*; FC = −1.75, *P*_adj_ < 0.014) (*30*) and *no blastema* (*nbl*/*hspd1*; FC = −1.32, *P*_adj_ < 0.001) (*31*), we compared the caudal fin transcriptomes with the recently described 49 evolutionarily conserved teleost regeneration genes (*13*). Of these 49 genes, 27 exhibited an impaired activation (FC ≥ ±1, *P*_adj_ ≤ 0.05) in *il11ra* mutants, while only 2 were up-regulated (Fig. 3, A and B, and table S3), at 24 hpa. In contrast, marker genes for different mature cell populations, including osteoblasts, epithelial, and mesenchymal cells, which are normally down-regulated during cellular reprogramming, remained at elevated expression levels in *il11ra* mutant fins after amputation (fig. S6). After caudal fin amputation, mature osteoblasts (*bglap*^+^) down-regulate *osteocalcin*/*bglap* expression and migrate toward the amputation plane, contributing to blastema formation (*32*, *33*). Hence, we used the *Tg*(*bglap:GFP*) reporter line in *il11ra* mutant background to investigate cellular reprogramming in vivo. We found that *Tg*(*bglap:*GFP) expression in the mutant fins fails to down-regulate proximal to the amputation plane at 48 hpa (Fig. 3, C and D). In addition, *Tg*(*bglap:*GFP)^+^ cells in the mutant stumps rarely translocated to participate in regeneration (Fig. 3, C and E). Similarly, in *il11ra* and *stat3* mutant larvae, we observed a reduced activation of genes reported (*34*) to be induced during fin fold regeneration at 24 hpa (fig. S7). Together, these data show that Il-11 signaling promotes central aspects of cellular reprogramming during fin regeneration.

Moreover, in the adult heart, we found several key regulators of zebrafish cardiac regeneration (*35*) to be dysregulated in *il11ra* mutants. These genes encode, among others, components of the complement cascade, retinoic acid signaling, the proregenerative ECM component Fibronectin (*fn1b*), and the injury-induced embryonic epicardial protein Wt1b (Fig. 3F). Notably, we also found that *myostatin* (*mstnb*), a negative regulator of cardiac regeneration, which is typically down-regulated after zebrafish cardiac cryoinjury (*16*), showed higher expression levels in the mutants compared with wild-type siblings (Fig. 3F). Furthermore, gene set enrichment (GSE) analysis and RT-qPCR experiments confirmed a substantially compromised transcriptional activation of retinoic acid metabolism (*aldh1a2*) in adult *il11ra* mutant hearts after injury (Fig. 3, G and H, and table S4). During adult zebrafish heart regeneration, *aldh1a2* is acutely activated organ-wide by endocardial and epicardial cells (*36*). Notably, immunostaining confirmed reduced endocardial-specific Aldh1a2 expression in *il11ra* mutants, while the epicardial expression remained comparable to that observed in wild types (Fig. 3I). Together, these data uncover a pivotal role for Il-11 signaling in cellular reprogramming and promoting important parts of the adult heart regeneration program in zebrafish.

**Fig. 4.**
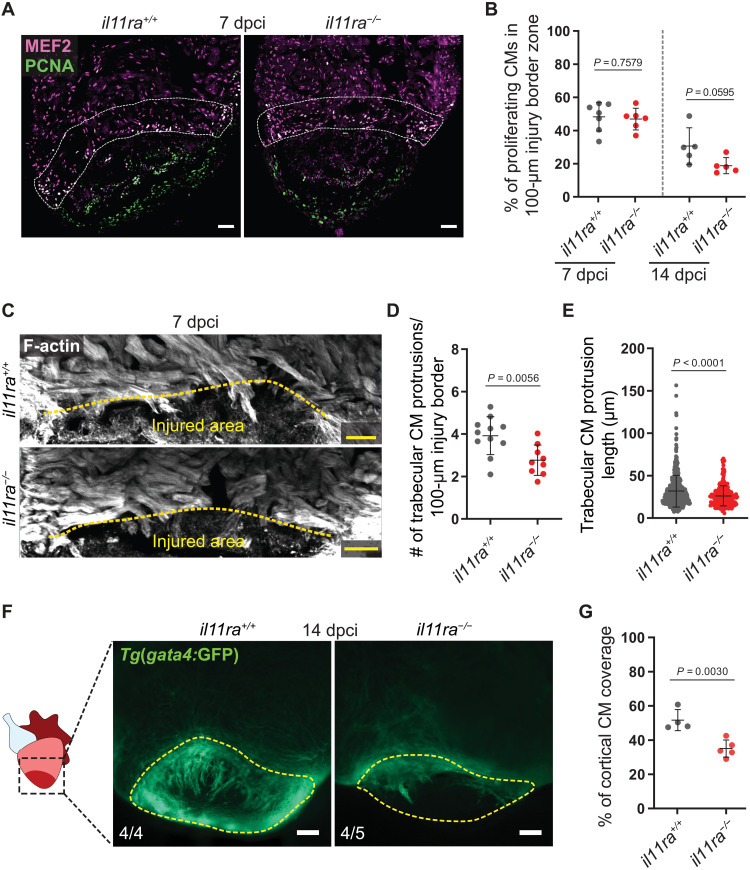
Il-11 signaling is required for CM repopulation after cardiac injury. (**A** and **B**) Costaining for MEF2 (magenta) and PCNA (green) expression to determine CM mitotic index on cryosections from ventricles (A) (wt siblings, *n* = 7; mut, *n* = 6; 7 dpci) and quantification within the 100-μm wound border zone for 7 and 14 dpci (B). (**C** to **E**) F-actin staining on 50-μm-thick cryosections from *il11ra^−/−^* versus wild-type ventricles, 7 dpci (C), and quantification of the number per ventricle (D) (wt siblings, *n* = 11; mut, *n* = 9) and length (E) (wt, *n* = 540; mut, *n* = 281) of CM protrusions. (**F** and **G**) Wholemount fluorescence images of *Tg*(*gata4:*EGFP) expression in ventricles (F) (wt siblings, *n* = 4; mut, *n* = 5; 14 dpci) and quantification of cortical CM wound coverage on the corresponding cryosections in fig. S8F (G). Data represent means ± SD (B, D, E, and G). Student’s *t* tests (B, D, and G); Mann-Whitney *U* test (E). *n*, ventricles (A, D, and F); *n*, CM protrusions (E). White dashed lines demarcate 100-μm injury border zone (A); yellow dashed lines demarcate the injured area (C and F). Scale bars, 50 μm (A and C) and 100 μm (F).

Fibronectin (Fn1) is deposited as a part of a transitional matrix in several tissues and species during regeneration (*37*–*40*). In line with our transcriptomic analyses, we observed a strong reduction in the Fibronectin-encoding gene *fn1b* expression, in the injured area of *il11ra* mutant hearts, adult fin stumps, and larval fin folds when compared with the respective wild-type siblings (Fig. 3J and fig. S7B). Moreover, using immunostaining, we confirmed reduced Fn1 deposition in the *il11ra* mutant ventricles in the injured area at 7 dpci (Fig. 3K). Fn1 deposition has been implicated in CM repopulation after cardiac injury in zebrafish (*39*). Hence, we assessed CM behavior after cryoinjury and found impaired trabecular CM protrusion in *il11ra* mutants at 72 hpci and 7 and 14 dpci (Fig. 4, C to E, and fig. S8, A to D), while CM proliferation was only affected at late stages (Fig. 4, A and B). To further investigate CM behavior, we used the *Tg*(*gata4:GFP*) reporter line. Upon injury, *Tg*(*gata4:*GFP) expression is up-regulated in border zone CMs, which are important contributors to the regenerate (*41*–*43*). While *Tg*(*gata4:*GFP) activation was comparable between *il11ra* mutants and wild types at 7 dpci (fig. S8E), coverage of the injured area by *Tg*(*gata4:*GFP)^+^ CMs was strongly reduced in the mutant hearts at 14 dpci (Fig. 4, F and G, and fig. S8F). These data suggest that Il-11 signaling is important for CM repopulation after cardiac injury. Together, the impaired activation of global and tissue-specific regeneration programs and impaired cell repopulation in *il11ra* mutants across tissues and developmental stages strongly indicate that Il-11 signaling acts as a high-level regulator, crucial to promote cellular reprogramming during tissue regeneration in zebrafish.

### Il-11/Stat3 signaling limits mammalian-like fibrosis after injury

**Fig. 5.**
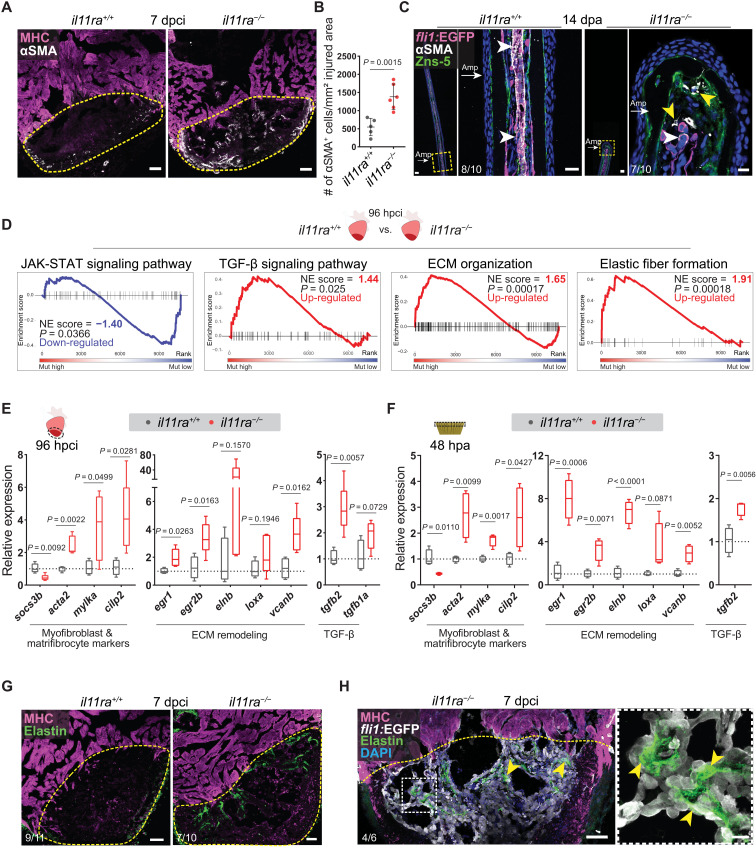
Il-11 signaling limits myofibroblast differentiation and profibrotic ECM remodeling after injury. (**A**) Immunostaining for αSMA (white) and MHC (magenta) expression on cryosections from cardiac ventricles (wt siblings, *n* = 5; mut, *n* = 6; 7 dpci). (**B**) Quantification of αSMA^+^ cell density. (**C**) Immunostaining for αSMA (white), GFP (magenta), and Zns-5 antigen (scleroblasts, green) expression on longitudinal cryosections from *Tg*(*fli1:EGFP*) caudal fins (wt, *n* = 10; mut, *n* = 10; 14 dpa). (**D**) GSE analysis plots for Reactome and KEGG pathway terms from *il11ra^−/−^* versus wild-type sibling adult ventricle transcriptomic analyses, 96 hpci. (**E** and **F**) RT-qPCR analysis on dissected injured areas from cardiac ventricles (E) (wt siblings, *n* = 4; mut, *n* = 5; 96 hpci) and caudal fins (F) (wt siblings, *n* = 4; mut, *n* = 4; 48 hpa) for selected profibrotic gene expression levels. (**G** and **H**) Immunostaining [(G) Elastin, green; MHC, magenta; (H) GFP, white] on cryosections from cardiac ventricles [(G) wt siblings, *n* = 11; mut, *n* = 10; (H) *Tg*(*fli1:EGFP*); mut, *n* = 6; 7 dpci]. Data represent means ± SD (B), and box plots (E and F) show median, interquartile range (IQR; box margins), and 5th and 95th percentiles (whiskers). Student’s *t* tests (B, E, and F). *n*, ventricles (A, E, G, and H); *n*, caudal fins (C); *n*, pools of two caudal fins (F). Yellow dashed lines demarcate the injured area (A, G, and H); yellow arrowheads point to αSMA^+^ myofibroblasts in *il11ra* mutant fins (C) and Elastin1 expression associated with endocardial cells in the injured area [inset in (H)]; white arrowheads point to vessel-associated αSMA^+^ smooth muscle cells (C); white arrows point to the amputation plane (C). Ct values are listed in table S5. Scale bars, 50 μm (A, C, G, and H), 20 μm [(C) left inset], and 10 μm [(C) right inset and (H) inset].

Canonical IL-11/STAT3 signaling has previously been described to be cardioprotective and antifibrotic in adult mammals (*44*, *45*). On the contrary, recent studies have reported that *Il11* expression is induced in the adult mouse heart after MI (*44*) and that noncanonical IL-11/ERK activity downstream of transforming growth factor–β (TGF-β) signaling is an important factor mediating fibrotic remodeling, proposing anti–IL-11 therapies to mitigate fibrosis (*46*, *47*). Specifically, inducing IL-11 increased while inhibiting IL11RA1 and ERK reduced myofibroblast differentiation and tissue fibrosis in mice. To investigate the role of Il-11 signaling in myofibroblast differentiation during regeneration in zebrafish, we quantified the number of αSMA^+^ myofibroblasts after cardiac cryoinjury. In line with the formation of a permanent scar at 90 dpci, *il11ra* mutants displayed a substantial increase in myofibroblasts at 7 dpci (Fig. 5, A and B), which was still present in the scars at 90 dpci (fig. S9, A and B). Notably, *il11ra* mutant caudal fin stumps also displayed myofibroblasts at 14 days after amputation (Fig. 5C), a time point at which wild-type fin regeneration is mostly complete. Consistent with these observations, GSE analysis revealed a substantial induction of TGF-β pathway components and ECM gene expression, as well as an impaired activation of Jak-Stat signaling pathway in *il11ra* mutant ventricles at 96 hpci (Fig. 5D and table S4). RT-qPCR analysis on the dissected injured areas of *il11ra* mutant and *stat3* heterozygous cardiac ventricles and *il11ra* mutant caudal fins further confirmed the induction of a multifaceted fibrotic gene program, including myofibroblast and matrifibrocyte markers, ECM components and remodeling enzymes, and profibrotic TGF-β ligands (Fig. 5, E and F, and fig. S9, C and D). Notably, *egr1* and *egr2b*, orthologs of key transcription factor genes expressed in mammalian tissue fibrosis downstream of TGF-β (*48*), displayed a robust induction in the nonregenerative *il11ra* mutant hearts and fins when compared with wild types (Fig. 5, E and F). Furthermore, in line with the induced *elnb* gene expression levels (Fig. 5E), immunostaining confirmed an increased deposition of Elastin1 in close proximity to endocardial cells in the mutant cardiac injured areas at 7 dpci (Fig. 5, G and H). To test whether this profibrotic response in zebrafish *il11ra* mutants is similar to that in nonregenerative adult mammals after cardiac injury, we used GSE analysis to identify the transcriptomic changes between *il11ra* mutant ventricles, as well as neonatal and adult mouse cardiac fibroblasts (*49*). Fibrosis-associated gene sets, including those encoding collagen biosynthesis and remodeling enzymes and regulators of epithelial-to-mesenchymal transition (EMT), were significantly enriched in both nonregenerative conditions but not in neonatal mouse cardiac fibroblasts (fig. S9, E and F, and table S4). Together, these analyses show that Il-11 signaling, in addition to promoting cellular reprogramming, limits maladaptive tissue remodeling in zebrafish, further emphasizing the negative correlation between myofibroblast differentiation and tissue regeneration.

### Il-11 signaling antagonizes EndoMT after cardiac injury

**Fig. 6.**
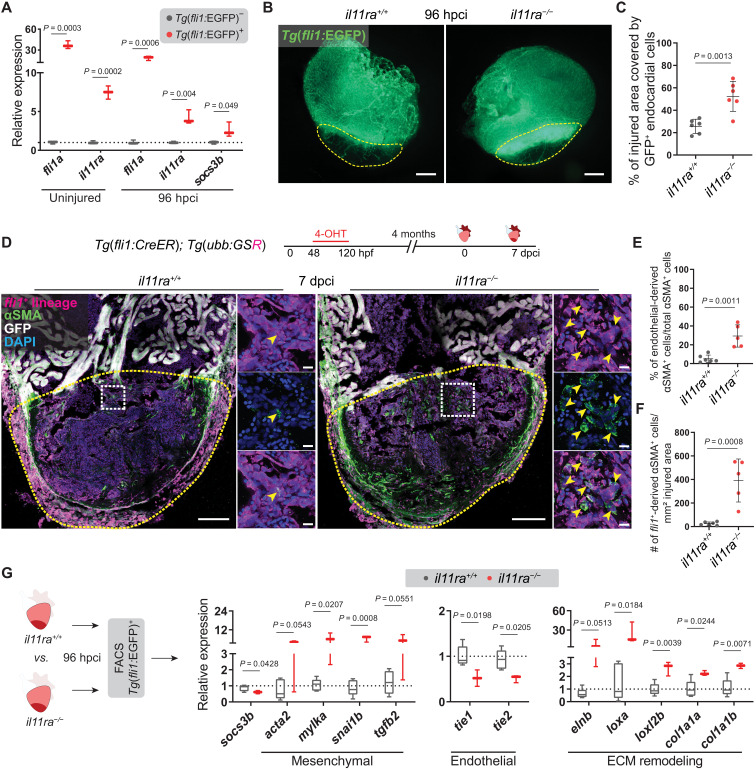
Il-11 signaling antagonizes EndoMT after cardiac injury. (**A**) RT-qPCR analysis on sorted *Tg*(*fli1:*EGFP)^+^ versus *Tg*(*fli1:*EGFP)^−^ cardiac ventricular cells (uninjured siblings, *n* = 3; 96 hpci, *n* = 3). (**B** and **C**) Wholemount fluorescence images of (B) (wt siblings, *n* = 6; mut, *n* = 6; 96 hpci) *Tg*(*fli1:*EGFP) expression in ventricles and quantification of percentage of injured area covered by GFP^+^ endocardial cells (C). (**D**) Experimental design and confocal images of immunostaining (mCherry, magenta; αSMA, green) on cryosections from (wt siblings, *n* = 6; mut, *n* = 5; 7 dpci) *Tg*(*fli1:CreER*)*; Tg*(*ubb:GSR*) ventricles. (**E** and **F**) Quantification of percentage (E) and density (F) of *fli1*^+^-derived αSMA^+^ cells in the injured area, 7 dpci. (**G**) Schematic and RT-qPCR analysis on sorted *Tg*(*fli1:*EGFP)^+^ cells from (wt siblings, *n* = 5; mut, *n* = 3; 96 hpci) ventricles for EndoMT-associated gene mRNA levels. Data represent means ± SD (C, E, and F), and box plots (A and G) show median, IQR (box margins), and 5th and 95th percentiles (whiskers). Student’s *t* tests (A, C, and E to G). *n*, pools of two ventricles (A and G), *n*, ventricles (B to F). Yellow dashed lines demarcate the injured area (B and D); yellow arrowheads point to *fli1*^+^-derived αSMA^+^ cells (D, insets). Ct values are listed in table S5. Scale bars, 200 μm (B), 100 μm (D), and 10 μm (D, insets).

To gain mechanistic insights into how Il-11 signaling regulates myofibroblast differentiation and ECM remodeling at the cellular level, we first determined the cell types expressing *il11ra* using available single-cell RNA-seq datasets (*50*, *51*). We found that, similar to its mammalian orthologs, zebrafish *il11ra* is highly expressed in epithelial and mesenchymal cells, including cardiac endothelia (fig. S10). Confirming these data, *il11ra* mRNA levels were higher in sorted endothelial cells from uninjured and 96-hpci wild-type ventricles when compared with the respective nonendothelial cells (Fig. 6A). In addition, we observed that sorted endothelial cells at 96 hpci displayed higher *socs3b* mRNA levels as a proxy for elevated Stat3 signaling, when compared with nonendothelial cells (Fig. 6A). Immunostaining for pStat3 at 96 hpci confirmed that endocardial cells in the injured area lack Stat3 activation in *il11ra* mutants (fig. S11A). These data, together with the reduced endothelial-specific Aldh1a2 expression (Fig. 3I) and Elastin1 deposition patterns (Fig. 5H) observed in *il11ra* mutant ventricles, show that cardiac endothelial cells are one of the predominant target cell types of Il-11/Stat3 signaling after cardiac injury. Hence, we analyzed *il11ra* mutants for potential endothelial phenotypes after cardiac cryoinjury. We found that endocardial cells in *il11ra* mutants displayed a significant increase in invasion of the injured area at 96 hpci (mutants, 52.43 ± 13.38%; wild types, 25.62 ± 6.35%) and 7 dpci when compared with wild types (Fig. 6, B and C, and fig. S11, B and C). Notably, at 7 dpci, endocardial cells in the injured area in *il11ra* mutants exhibited a disorganized architecture unlike the cohesive network observed in wild types (fig. S11B). This hyper-invasive endothelial phenotype, together with increased myofibroblast differentiation and profibrotic remodeling in *il11ra* mutants, led us to hypothesize that Il-11 signaling limits endothelial-to-mesenchymal transition (EndoMT) during adult zebrafish heart regeneration.

EndoMT is an EMT-like process in which endothelial cells gain mesenchymal-like properties, contributing to diverse types of organ fibrosis, including in the heart (*52*). To test for EndoMT, we investigated the expression of several EndoMT-associated genes in dissected injured areas from adult *il11ra* mutants, *stat3* heterozygotes, and their respective wild-type siblings at 96 hpci. Mesenchymal marker genes—including *acta2*, *mylka*, *sox9a*, and *vcanb*, as well as EndoMT-inducing factor genes such as *tgfb2*, *edn1*, and *snai1b*—displayed significant up-regulation in *il11ra* mutants and a similar trend in *stat3* heterozygotes (fig. S11, D and E). To confirm EndoMT, we lineage traced *fli1^+^* endothelial cells and immunostained for the myofibroblast markers, αSMA and myosin light chain kinase (MLCK) (*10*). Consistent with our earlier endothelial lineage tracing experiments (Fig. 1B), wild types displayed rather low proportions (5.13 ± 3.97%) of EndoMT in the injured area at 7 dpci (Fig. 6, D and E). Notably, *il11ra* mutants displayed significantly elevated levels of EndoMT (29.34 ± 11.84%) (Fig. 6, D to F). In addition, we observed that endothelial-derived MLCK^+^ cells in the injured area in *il11ra* mutants exhibited reduced Cdh5 membrane localization at 7 dpci (fig. S11F), further confirming EndoMT. To analyze whether the increased EndoMT contributes to fibrotic ECM remodeling, we sorted endothelial cells from *il11ra* mutant and wild-type ventricles at 96 hpci (Fig. 6G). RT-qPCR analysis revealed an up-regulation of mesenchymal gene expression along with a down-regulation of endothelial gene expression and Stat3 activation in the endothelial cells sorted from the mutants when compared with wild types (Fig. 6G). Furthermore, several profibrotic ECM remodeling genes were significantly up-regulated in the mutant endothelial cells (Fig. 6G). In addition, since *il11ra* is also expressed in the epicardium (fig. S10A), we lineage traced epicardial cells to determine their contribution to myofibroblasts in *il11ra* mutants. While the density of epicardial-derived cells in the injured area remains unchanged when compared with wild types at 7 dpci, *il11ra* mutants display a significantly elevated number of epicardial-derived myofibroblasts when compared to wild types at 7 dpci (fig. S12). Thus, these data further substantiate the observations that Il-11 signaling is antifibrotic in both endothelial and epicardial lineages and limits mammalian-like scarring during adult heart regeneration in zebrafish.

### Il-11 signaling in endothelial cells allows CM repopulation after cardiac injury

**Fig. 7.**
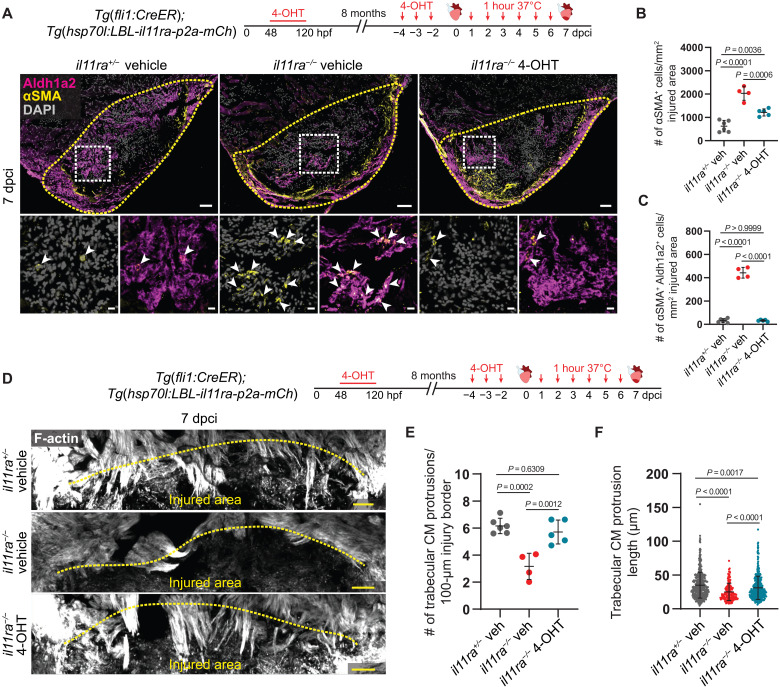
Il-11 signaling in endothelial cells allows CM repopulation after cardiac injury. (**A**) Experimental design and confocal images of immunostaining (Aldh1a2, magenta; αSMA, yellow) on cryosections from vehicle-treated *il11ra^+/−^* siblings (*n* = 6) and vehicle- or 4-OHT–treated *il11ra^−/−^* (*n* = 4 and 5, respectively) *Tg*(*fli1:CreER*)*; Tg*(*hsp70l:LBL-il11ra-p2a-mCh*) ventricles at 7 dpci. (**B** and **C**) Quantification of total αSMA^+^ cell density (B) and αSMA^+^ Aldh1a2^+^ cell density (C) in the injured area, 7 dpci. (**D**) Experimental design and F-actin staining on 50-μm-thick cryosections from vehicle-treated *il11ra^+/−^* siblings and vehicle- or 4-OHT–treated *il11ra^−/−^ Tg*(*fli1:CreER*)*; Tg*(*hsp70l:LBL-il11ra-p2a-mCh*) ventricles at 7 dpci. (**E** and **F**) Quantification of the number per ventricle (E) (*il11ra^+/−^* veh, *n* = 6; *il11ra^−/−^* veh, *n* = 4; *il11ra^−/−^* 4-OHT, *n* = 5) and length (F) (*il11ra^+/−^* veh, *n* = 481; *il11ra^−/−^* veh, *n* = 185; *il11ra^−/−^* 4-OHT, *n* = 437) of CM protrusions at 7 dpci. Data represent means ± SD (B, C, E, and F). One-way ANOVA (B, C, and E); Kruskal-Wallis test (F). *n*, ventricles (A and E); *n*, CM protrusions (F). Yellow dashed lines demarcate the injured area (A and D); white arrowheads point to αSMA^+^ Aldh1a2^+^ endocardial cells (A, insets). Scale bars, 50 μm (A and D) and 10 μm (A, insets).

Secreted factors, including ECM proteins, have been shown to strongly influence CM regeneration after injury (*53*–*56*). *il11ra* mutant hearts, after injury, display EndoMT-mediated fibrosis, a lack of transitional matrix deposition, and CM repopulation defects. Hence, we aimed to determine whether reduced Il-11 signaling in endothelial cells was a contributor to the observed CM repopulation defects. To address this question, we used the HOTcre system (*57*) and generated a transgenic line, *Tg*(*hsp70l:LBL-il11ra-p2a-mCherry*), that conditionally reexpresses *il11ra* under the *hsp70l* promoter. Using this line in combination with the endothelial *Tg*(*fli1:CreER*) line allows spatial and temporal control over the endothelial-specific reexpression of *il11ra-p2a-mCherry*. We treated these double transgenic fish [*Tg*(*fli1:CreER*); *Tg*(*hsp70l:LBL-il11ra-p2a-mCherry*)] in *il11ra* heterozygous and homozygous mutant background with vehicle or 4-hydroxytamoxifen (4-OHT) (fig. S13A). Subsequently, we performed cardiac cryoinjury, followed by daily heat shocks from 1 to 6 dpci, and analyzed the hearts at 7 dpci. First, we tested the conditional reexpression of *il11ra-p2a-mCherry* by immunostaining for mCherry expression. We observed Cdh5^+^ endothelial cells expressing mCherry only in the 4-OHT–treated animals but not in the vehicle controls (fig. S13A). Next, we tested whether Il-11 signaling in endothelial cells could limit endothelial invasion of the injured area and EndoMT. We found that endothelial-specific reexpression of *il11ra* in *il11ra* mutant background rescued the hyper-invasive endocardial cell phenotype, which was reduced to heterozygous control levels (fig. S13, B and C). Moreover, endothelial-specific reexpression of *il11ra* in *il11ra* mutants significantly reduced αSMA^+^ myofibroblast density in the injured area when compared with vehicle-treated mutants but remained higher than that observed in the heterozygotes (Fig. 7, A and B). Endothelial-specific quantification showed that endocardial contribution to myofibroblast differentiation in the rescued mutants was minimal and equivalent to heterozygous control levels (Fig. 7C), suggesting that the remaining cortically localized myofibroblasts in the rescued mutants are likely epicardial derived. Together, these data show that injury-induced Il-11 signaling in endothelial cells can limit EndoMT. Furthermore, to investigate whether Il-11 signaling in endothelial cells was important for CM repopulation of the injured area, we quantified CM protrusions. Strikingly, we found that endothelial-specific reexpression of *il11ra* in *il11ra* mutants restored CM protrusions almost to the heterozygous control levels (Fig. 7, D to F). Together, these data show that injury-induced Il-11 signaling in endothelial cells can limit EndoMT-mediated fibrosis and promote CM repopulation in *il11ra* mutants.

### Endothelial IL-11 signaling feeds back to inhibit TGF-β signaling

**Fig. 8.**
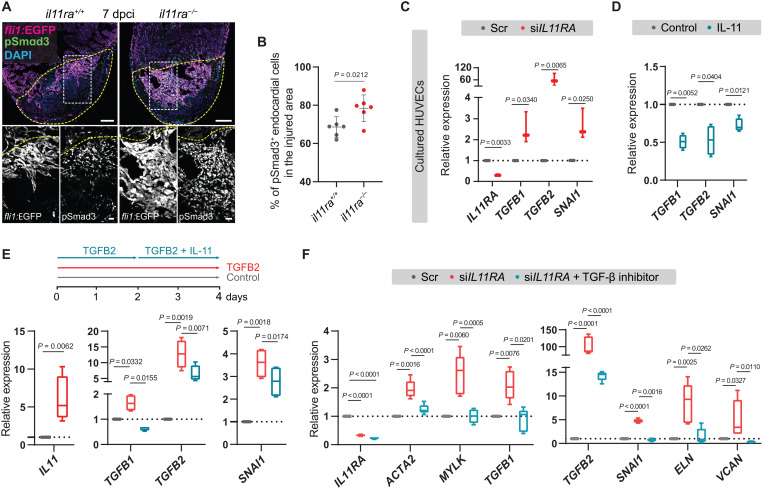
IL-11 signaling feeds back to inhibit TGF-β–mediated scarring. (**A** and **B**) Confocal images of immunostaining (A) (GFP, magenta; pSmad3, green; 7 dpci) on cryosections from *il11ra^−/−^* versus wild-type *Tg*(*fli1:EGFP*) ventricles and quantification of percentage of pSmad3^+^ endocardial cells in the injured area (B) (wt siblings, *n* = 6; mut, *n* = 6). (**C**) RT-qPCR analysis for *IL11RA*, genes encoding TGF-β ligands, and TGF-β downstream target SNAI1 mRNA levels on HUVECs transfected with scrambled (*n* = 3) or *IL11RA* siRNAs (*n* = 3). (**D**) RT-qPCR analysis for genes encoding TGF-β ligands and for TGF-β downstream target SNAI1 mRNA levels on HUVECs treated with control (*n* = 4) or rhIL-11 (10 ng/ml; *n* = 4). (**E**) Experimental design and RT-qPCR analysis for *IL11*, genes encoding TGF-β ligands, and for TGF-β downstream target SNAI1 mRNA levels on HUVECs treated with control (*n* = 4) or rhTGFB2 (10 ng/ml; *n* = 4) or rhTGFB2 + rhIL-11 (10 ng/ml) (*n* = 4). (**F**) RT-qPCR analysis for genes encoding myofibroblast markers, TGF-β ligands, TGF-β downstream target SNAI1, and for fibrogenic ECM component mRNA levels on HUVECs transfected with scrambled (*n* = 4) or si*IL11RA* (*n* = 4) or si*IL11RA* + 10 μM TGFBR1 inhibitor (SB431542; *n* = 4). Data represent means ± SD (B); box plots (C to F) show median, IQR (box margins), and 5th and 95th percentiles (whiskers). Student’s *t* tests [(B) to (D) and (E) *IL11*]; one-way ANOVA (E and F). *n*, ventricles (B); *n*, biological replicates (C to F). Yellow dashed lines demarcate the injured area (A). Ct values are listed in table S5. Scale bars, 100 μm (A) and 20 μm (A, insets).

TGF-β signaling is a key regulator of tissue fibrosis, including EndoMT, acting through a signaling cascade that results in the phosphorylation and activation of SMAD transcription factors (*52*). To test whether the high levels of TGF-β ligand expression observed in endothelial cells in *il11ra* mutant ventricles (Fig. 6G) after injury resulted in increased SMAD activation, we immunostained for pSmad3 in the endothelial *Tg*(*fli1:EGFP*) line. We observed that *il11ra* mutants display a significantly higher proportion of pSmad3^+^ endocardial cells in the injured area at 7 dpci (mutants, 78.43 ± 6.97%; wild types, 68.63 ± 5.36%), further confirming that *il11ra* mutants display elevated endothelial TGF-β signaling activity after cardiac injury (Fig. 8, A and B). To investigate a possible interaction between the IL-11 and TGF-β signaling pathways, we used primary human umbilical vein endothelial cells (HUVECs) in culture. In line with a previous report in fibroblasts (*46*), we found that TGF-β stimulation induced *IL11* expression in HUVECs (Fig. 8E). Next, to test whether IL-11 acts in a negative feedback mechanism as suggested by our in vivo data, we knocked down (KD) *IL11RA* using small interfering RNA (siRNA) and treated HUVECs with IL-11. We observed that KD of *IL11RA* alone was sufficient to induce *TGFB2* (59-fold), *TGFB1* (2.5-fold), and SMAD target gene *SNAI1* (2.7-fold) expression (Fig. 8C) when compared with scrambled controls and that IL-11 treatment robustly down-regulated the same genes (Fig. 8D). Furthermore, treating TGFB2-stimulated cells with IL-11 significantly reduced *TGFB2*, *TGFB1*, and *SNAI1* expression compared with TGFB2 stimulation alone (Fig. 8E). In line with inducing TGF-β signaling (Fig. 8C), knocking down *IL11RA* also activated fibrogenic gene expression (Fig. 8F). In addition, we tested whether this profibrotic gene expression could be rescued by blocking TGF-β signaling. Blocking TGFBR1/ALK5 function, by using the small-molecule inhibitor SB431542, rescued myofibroblast marker and fibrogenic ECM gene expression in *IL11RA* KD cells (Fig. 8F). Together, these data reveal an important negative feedback mechanism in which IL-11 signaling limits scarring by antagonizing TGF-β signaling in endothelial cells. Overall, from these in vivo and in vitro findings, we conclude that IL-11 signaling antagonizes cardiac tissue fibrosis after injury, at least in part by negatively regulating its activator, TGF-β signaling.

## DISCUSSION

Regeneration and permanent scarring are two opposing end points in response to tissue damage. In regenerative species, cells of a damaged tissue undergo reprogramming, including a switch in their individual transcriptional profiles away from a homeostatic program, toward establishing a regenerative niche. In this process, genes not needed or detrimental to regeneration become transcriptionally silenced, while others become activated to promote the formation of a regenerate, as shown in the axolotl limb (*15*) and the zebrafish fin and heart (*35*, *51*). In contrast, most adult mammals, including humans, predominantly induce a fibrogenic program in response to injury. This program commonly leads to myofibroblast differentiation, mostly derived from tissue-resident fibroblasts and endothelial cells, resulting in excessive matrix deposition and the secretion of ECM cross-linking enzymes (*2*). Ultimately, this fibrotic tissue remodeling establishes a functionally inert scar and limits regeneration. Consequently, a wealth of studies have aimed to identify mechanisms that limit fibrotic scarring or promote regeneration to develop strategies for regenerative medicine. These studies have led to several important discoveries, including the identification of many components of the regeneration program, acting either tissue-specifically or globally. However, most of these signaling pathways are indispensable during development. Hence, the existence of regeneration-specific global regulators remains elusive.

In this study, we addressed this problem by performing a comparative expression profiling under physiological and pathological conditions using the regenerating zebrafish heart as a model. We identify Il-11, signaling through its receptor Il11ra, as an injury-responsive signal, largely dispensable for development but globally essential for regeneration in zebrafish. Mechanistically, we show that Il-11 signaling is essential for cellular reprogramming to activate global and tissue-specific regenerative programs. Moreover, we show that Il-11 signaling inhibits a mammalian-like scarring response across diverse tissues in zebrafish.

Blastema formation, following injury, is a hallmark of diverse regenerative species and tissues, including the zebrafish fins, axolotl limbs, *Xenopus* tadpole tails and limbs, mouse digit tips, and spiny mouse ears. The blastema is a progenitor cell mass that has been shown to form predominantly by cellular reprogramming, providing a regenerative niche and contributing to rebuilding the lost tissue. In axolotl, during limb blastema formation, it was reported that connective tissue cells lose their mature features and acquire a similar identity (*15*). Likewise, during adult zebrafish caudal fin regeneration, osteoblasts dedifferentiate and migrate, contributing to blastema formation (*32*, *33*). Expression profiles from the axolotl limb (*15*) and *Xenopus* tadpole tail (*26*) regenerates show that *Il11* is transiently induced, suggesting a potential role for Il-11 signaling in cellular reprogramming. Gene KD studies in the regenerating *Xenopus* tadpole tail indicate that Il-11 signaling is important to induce and maintain the blastema (*26*). Consolidating these findings, our work in the adult zebrafish caudal fin now provides genetic evidence that compromised Il-11 signaling results in severe defects in blastema formation, cellular reprogramming, and the induction of blastema-specific regenerative genes. Similarly, we show that Il-11 signaling also promotes cellular reprogramming in the adult zebrafish heart after injury. However, the existence of blastema-like features in the regenerating heart has not been described thus far and will need further investigation.

Defects in cellular reprogramming culminate in severely compromised regeneration across tissues. We found that repopulation of injured area as evident by translocation of regenerating cells to the site of damage is largely affected in *il11ra* mutant hearts and fins. For the heart, *il11ra* mutants display severely compromised CM protrusion starting at 72 hpci. CMs, however, are not the principal expression domain of *il11ra*, which is predominantly expressed in nonmyocardial cells, including endothelial cells and fibroblasts, an expression pattern also reported in other species, including mammals (*46*). We show that cardiac endothelial Stat3 activation is severely reduced in *il11ra* mutants at 96 hpci. Moreover, endothelial-specific reexpression of *il11ra* in the mutant hearts rescued CM protrusion of the injured area. Together, these data strongly argue that the CM protrusion defects are possibly due to cell nonautonomous effects, including the lack of promigratory matrix deposition, and EndoMT-mediated excessive scar matrix deposition. Unexpectedly, we did not observe any CM proliferation defects in *il11ra* mutants at 7 dpci but only a mild reduction at 14 dpci. In line with this, previous work in the regenerating zebrafish heart showed that blocking Fn1 deposition did not affect CM proliferation at 7 dpi but resulted in impaired CM repopulation at 30 dpi (*39*). Other work in mice highlights the importance of matrix composition in regulating CM regeneration (*53*–*55*). With these data, we speculate that CM proliferation defects at 14 dpci in *il11ra* mutants are secondary to the lack of a permissive microenvironment. However, further cell type–specific rescue experiments will be needed to conclusively analyze the effects of stromal cell Il-11 signaling during regeneration. Together, with our findings, these data suggest a conserved and most central function for Il-11 signaling in cellular reprogramming during organ, appendage, and more general tissue regeneration in regenerative species, including tetrapods.

While mammals predominantly respond to tissue damage with scarring, regenerative species only exhibit limited scar formation. Using lineage tracing, we show that the regenerating zebrafish heart displays only minor myofibroblast differentiation from epicardial- and endothelial-derived lineages, when compared to adult mice after MI. These proportions fundamentally change in *il11ra* mutant heart and fins. For the heart, epicardial- and endothelial-derived lineages both display a significant transdifferentiation toward a myofibroblast fate in *il11ra* mutants. FAP (fibroblast activation protein), a recently reported marker for activated fibroblasts (*6*), along with 9 of the top 15 matrifibrocyte marker genes (*3*), displays elevated expression levels in *il11ra* mutant hearts after injury. In line with this observation, *il11ra* mutants display increased expression of key regulators of mammalian tissue fibrosis (*egr1*, *egr2b*, and *meox1*), as well as the secretion of fibrotic ECM components and cross-linking enzymes, after injury. Notably, MEOX1 has recently been reported as a transcriptional switch for fibroblast activation during cardiac fibrosis in mammals (*58*). Unbiased GSE analysis underlines that *il11ra* mutant hearts share important aspects of fibrosis with the nonregenerative adult mouse hearts, but not with the regenerative neonatal hearts, after injury. Together, these data strongly indicate that Il-11/Stat3 signaling limits hallmarks of the mammalian scarring program and promotes central aspects of global tissue regeneration in zebrafish.

The role of IL-11 signaling in mammals after tissue damage is highly debated. Recombinant human (rh) IL-11, a Food and Drug Administration (FDA)–approved drug for treating thrombocytopenia (*59*), was initially claimed to be cardioprotective and antifibrotic (*44*, *45*). These reports showed that injecting rhIL-11 ameliorated fibrosis and promoted cardiac function after MI in adult mouse. Following these promising rodent studies, human MI patients were administered with rhIL-11 in an investigational therapy, with no adverse reactions observed (*60*). In line with these reports, an engineered version of IL-6 (hyper IL-6), which, similar to IL-11, signals through GP130 to activate STAT3, was recently used successfully to promote central nervous system regeneration in adult mice (*61*). In contrast, increased IL-11 levels in patients with chronic heart failure were reported to correlate with cardiac events (*62*). Moreover, other recent studies have reported a profibrotic role for IL-11 in diverse tissues, portraying it as a key regeneration-limiting and fibrogenic molecule (*46*, *47*). These studies show that TGF-β transcriptionally activates IL-11, which then drives fibrogenic protein synthesis through noncanonical ERK activity in fibroblasts. Furthermore, it was also reported that blocking IL-11 signaling protects from maladaptive remodeling after a range of profibrotic stimuli. Hence, these studies proposed antibody-based IL-11 antagonism therapies to reverse inflammation and fibrosis and restore organ function.

While our results in zebrafish clearly support the antifibrotic and proregenerative side of the debate, some critical aspects need to be carefully considered. First, the evolutionary changes in the components of IL-11 signaling: Our phylogenetic analyses and the conserved synteny confirm that the zebrafish Il11ra and Il-11 encoding genes investigated in this study are direct orthologs of the human IL11RA and IL-11 encoding genes. Notably, mice, unlike zebrafish and humans, carry a duplication of *Il11ra* (*Il11ra1* and *Il11ra2*), which could potentially complicate a mechanistic analysis. Nevertheless, IL-11 signaling might have drifted away from controlling a regenerative gene program through canonical STAT3 to predominantly inducing fibrotic scarring through the noncanonical ERK signaling pathway in mammals. This hypothesis is further supported by several other mammalian studies that have reported a proregenerative role for the components of IL-6 family/STAT3 signaling after tissue damage (*27*, *63*–*66*). Furthermore, in the zebrafish heart, we found that endocardial Stat3 activation after injury depended on Il-11 signaling, while in mammals, it was reported that stimulation with IL-11 only resulted in a transient Stat3 activation for various cell types in vitro (*67*, *68*). This hypothesis may explain in part why nonregenerative species do not form a blastema after injury, but in contrast predominantly display myofibroblast activation and scar formation, highlighting the need for a deeper comparative investigation of the mechanisms downstream of IL11RA/Il11ra activation in regenerative and nonregenerative species. Despite these potential evolutionary differences, our analyses in primary human endothelial cells in culture uncover a conserved negative feedback interaction in this cell type between the IL-11 and TGF-β signaling pathways. Notably, our zebrafish and human data show the antifibrotic and proregenerative effects of IL-11 signaling mainly in endothelial cells, while the profibrotic role in mammals was established in fibroblasts (*46*). However, in the zebrafish heart after injury, our lineage tracing data indicate that *il11ra* function also limits myofibroblast differentiation from the epicardial-derived fibroblast lineage. These data highlight the importance of studying the cell type–specific roles of IL-11 signaling during regeneration and scarring.

Together, it is now clear that a deeper understanding of potentially fundamental differences downstream of IL-11 signaling between regenerative and nonregenerative species and between different cell types will be of utter importance to develop regenerative and antifibrotic therapies. In addition, the homozygous viable, nonregenerative *il11ra* zebrafish mutant, with its mammalian-like scarring phenotype after injury, offers exciting new possibilities to serve as a model for mammalian fibrosis and to investigate antifibrotic and proregenerative mechanisms.

## MATERIALS AND METHODS

### Zebrafish handling

All zebrafish (*Danio rerio*, strain: Tüb/AB) husbandry was performed under standard conditions in accordance with institutional (Max-Planck-Gesellschaft) and national ethical and animal welfare guidelines approved by the ethics committee for animal experiments at the Regierungspräsidium Darmstadt, Germany.

### Zebrafish lines

The following transgenic and mutant lines were used in this study: *TgBAC*(*cryaa:EGFP,tcf21:Cre-ERT2*)*pd42* (*69*), abbreviated as *Tg*(*tcf21:CreER*); *Tg*(*kdrl:Cre*)*s898* (*70*), abbreviated as *Tg*(*kdrl:Cre*); *Tg*(*fli1:Cre-ERT2*)*cn9* (*12*), abbreviated as *Tg*(*fli1:CreER*); *Tg*(*-3.5ubb:LOXP-EGFP-LOXP-mCherry*)*cz1701* (*71*), abbreviated as *Tg*(*ubb:GSR*); *Tg*(*-3.5ubb:LOXP-LacZ-LOXP-egfp*)*cn2* (*72*), abbreviated as *Tg*(*ubb:laczSG*); *Tg*(*-14.8gata4:GFP*)*ae1* (*73*), abbreviated as *Tg*(*gata4:GFP*); *Tg1*(*Ola.Bglap:EGFP*)*hu4008* (*74*), abbreviated as *Tg*(*bglap:GFP*); *Tg*(*fli1:EGFP*)*y1* (*75*), abbreviated as *Tg*(*fli1:EGFP*); *ET*(*krt4:EGFP*)*sqet33-1A* (*76*), abbreviated as *Tg*(*ET33:EGFP*); *Tg*(*hsp70l:loxp-lox2272-mCherry-loxp-il11ra-V5-p2a-GFP-lox2272*)*bns546* (this study); *Tg*(*hsp70l:loxP-TagBFP-loxP-il11ra-t2A-mCherry*)*bns417* (this study), abbreviated as *Tg*(*hsp70l:LBL-il11ra-p2a-mCh*); *stat3^stl27^* (*22*), *il6st^sa1462^* (*77*), *il11ra^bns251^* (this study), *il11a^bns311^* (this study), and *il11b^bns312^* (this study).

### Generation of zebrafish transgenic and mutant lines

CRISPR-Cas9 technology was used to generate *il11ra^bns251^*, *il11a^bns311^*, and *il11b^bns312^*. The following guide RNA (gRNA) sequences (5′-3′) were used: ATGGTGGAGTTAGATCCCA**CGG** (exon 6–*il11ra*), GTACAGAGATTAATCATCAC**CGG** (exon 3–*il11a*), and TCCGTTGGACCCAATCAAGA**TGG** (exon 3–*il11b*), respectively.

Fifty picograms (pg) of individual gRNAs together with 150 pg of Cas9 mRNA was injected into zebrafish embryos at the one-cell stage. Mutant alleles were identified by high-resolution melt analysis. Predicted resulting peptides for all the mutant alleles are illustrated in Fig. 2 and figs. S2 and S3. Primer sequences used for genotyping are as shown in table S5.

To generate the *hsp70l:loxp-lox2272-mCherry-loxp-il11ra-V5-p2a-GFP-lox2272* construct, *hsp70l* promoter from the HOTCre construct (*57*), *loxp* and *lox2272* sequences from *ubb:lox2272-mCerulean-UAS-loxP-lox2272-GAL4-loxP-LIFEACT-GFP* (*78*), *mCherry*, and *il11ra-V5-p2a-GFP* were individually amplified and sequentially cloned into miniTol2 vector. *il11ra* coding sequence was amplified from complementary DNA (cDNA) reverse-transcribed from adult zebrafish caudal fin total RNA. To generate the *hsp70l:loxP-TagBFP-loxP-il11ra-t2A-mCherry* construct, the HOTCre plasmid (*57*) (*hsp70l:loxP-mCherry-STOP-loxP-H2B-GFP; cryaa:Cerulean*) was modified as follows. First, *mCherry* was replaced by *TagBFP*, and *H2B-GFP* was replaced by *il11ra-t2A-mCherry*. We then amplified *hsp70l:loxP-TagBFP-loxP-il11ra-t2A-mCherry* and cloned it into miniTol2 vector. To generate the transgenic lines *Tg*(*hsp70l:loxp-lox2272-mCherry-loxp-il11ra-V5-p2a-GFP-lox2272*)*bns546* and *Tg*(*hsp70l:loxP-TagBFP-loxP-il11ra-t2A-mCherry*)*bns417*, one-cell–staged zebrafish embryos were injected with the constructs *hsp70l:loxp-lox2272-mCherry-loxp-il11ra-V5-p2a-GFP-lox2272* and *hsp70l:loxP-TagBFP-loxP-il11ra-t2A-mCherry*, respectively, together with 50 pg of Tol2 mRNA. The injected embryos were grown to adulthood and screened for germline transmission using heat shock–dependent mCherry and TagBFP expression, respectively. To induce recombination, one-cell–stage *Tg*(*hsp70l:loxp-lox2272-mCherry-loxp-il11ra-V5-p2a-GFP-lox2272*) embryos were injected with 5 pg (per embryo) of Cre mRNA.

### Cardiac cryoinjury

Adult zebrafish [4 to 8 months post fertilization (mpf)] were anesthetized in 0.016% tricaine in system water and placed on a wet sponge with their ventral side up. An incision was made through the chest to access the heart, and a cryoprobe precooled with liquid nitrogen was applied to the ventricular apex until the cryoprobe thawed. Later, the fish were allowed to recover by transferring them into fresh system water.

### Adult caudal fin and larval fin fold injuries

Adult zebrafish (4 to 8 mpf) were anesthetized in 0.016% tricaine in system water, and the caudal fins were amputated under a stereomicroscope using a scalpel, or five to six individual fin rays were crushed gently using forceps. The fish were allowed to recover in fresh system water, and fins were allowed to regenerate at 28°C, until the indicated time points. Caudal fin tissue, two bone segments proximal to the amputation plane, was collected for gene expression analyses. Larval zebrafish at 48 to 72 hours post fertilization were anesthetized in 0.016% tricaine in egg water, and their fin folds posterior to the notochord were amputated under a stereomicroscope using a scalpel. The fin folds were allowed to regenerate at 28°C until the indicated time points. For gene expression analysis, tissue posterior to the yolk extension was collected.

### Adult scale injury

Adult zebrafish (4 to 8 mpf) were anesthetized in 0.016% tricaine in system water. Subsequently, they were placed on a petri dish lid under a stereomicroscope. Approximately 10 scales, 3 to 4 each from three rows on the side of the body posterior to the pectoral fins, were removed with forceps. The fish were then allowed to recover in fresh system water.

### Zebrafish exercise training

Exercise training was performed as described (*79*). In short, adult zebrafish (6 to 8 mpf) were placed in a 5-liter glass beaker filled with 4 liters of system water. A stream was generated with a magnetic stir bar, which induced swimming behavior against the stream, simulating exercise. Fish were trained for two times 4 hours a day with 1 hour of rest in between, for 5 days in a row. Exercised fish and the corresponding controls (without stir bar) were then sacrificed on day 5 to harvest their ventricles for gene expression analysis.

### Tamoxifen treatment

Zebrafish embryos and larvae were treated with 5 μM 4-OHT (H7904, Sigma-Aldrich) dissolved in pure ethanol (25 mM stock) and diluted in egg water at 28°C for time periods as mentioned in the respective figures. 4-OHT stock was preheated at 60°C for 10 min before diluting in egg water. Adult fish were injected intraperitoneally with 10 μl of 1.25 mM 4-OHT or 5% ethanol as a vehicle control, diluted in sterile 1× phosphate-buffered saline (PBS).

### Histological analysis and imaging

The hearts and fins were fixed using PEM fixative [3% paraformaldehyde (PFA), 100 mM Pipes, 1 mM MgSO_4_, and 2 mM EGTA in distilled water and adjusted to pH 7.4] for 1 hour at room temperature on a nutator. The tissues were cryopreserved overnight (O/N) at 4°C in 30% (w/v) sucrose solution prepared in 1× PBS. The hearts were then embedded in O.C.T. (optimal cutting temperature) compound (Tissue-Tek) and stored at −80°C. The adult caudal fins were preembedded in 7.5% (w/v) porcine gelatin (Sigma-Aldrich)/15% (w/v) sucrose in 1× PBS at 37°C for 1 hour and embedded with a new solution of gelatin. Fin tissue blocks were gradually frozen in isopentane (Sigma-Aldrich) cooled in liquid nitrogen. Cryosections (11 and 50 μm thick) were collected on SuperFrost Plus (Thermo Fisher Scientific) slides using Leica CM1950 cryostat and stored at −20°C.

For AFOG staining, cryosections were fixed with Bouin’s solution for 2 hours at 60°C and stained according to the manufacturer’s instructions (AFOG staining kit, BioGnost), without hematoxylin solution. Imaging was performed using Nikon SMZ25 or Zeiss widefield (Axio Imager) microscopes.

To perform immunofluorescence staining, O.C.T. was removed from 11-μm-thick cryosections by rinsing the slides with 1× PBS. To remove gelatin from the fin cryosections, slides were rinsed with 1× PBS at 37°C for 10 min. The sections were then permeabilized with 0.5% Triton X in 1× PBS for 20 min (2 hours for 50-μm cryosections) at room temperature followed by incubation in blocking buffer [1× PBS, 2% (v/v) donkey serum, 0.2% Triton X-100, and 1% dimethyl sulfoxide] for 1 hour at room temperature. Later, the sections were incubated with primary antibodies in blocking buffer O/N at 4°C with parafilm coverslips for even distribution. After washing for at least 2 hours, the sections were incubated with secondary antibodies in blocking buffer for 3 hours at room temperature. Last, the immunostained slides, after washing and staining with 4′,6-diamidino-2-phenylindole (DAPI; 1:10,000, 10 mg/ml stock; Sigma-Aldrich), were mounted with fluorescence mounting medium (S3023, Agilent Dako) for imaging. Mef2, PCNA, and pSTAT3 immunostaining was performed as described earlier (*42*). Imaging was performed using Zeiss LSM 800 Observer or inverted Zeiss Cell Observer SD confocal microscopes. Nikon SMZ25 was used for wholemount ventricle (fluorescence and bright-field), adult fin, and larval fin fold imaging.

Primary antibodies used for immunofluorescence were as follows: anti-pSTAT3 Y705 at 1:100 (rabbit, 9131s, Cell Signaling Technology), anti-Mef2 at 1:100 (rabbit, sc-313, Santa Cruz), anti-PCNA at 1:100 (mouse; sc-56, Santa Cruz), anti–myosin heavy chain (MHC) at 1:200 (mouse, MF-20, Developmental Studies Hybridoma Bank), anti-αSMA at 1:100 (rabbit, GTX124505, GeneTex), anti–green fluorescent protein (GFP) at 1:200 (chicken, GFP-1010, Aves Labs), anti-Elastin1 (*80*) at 1:100 (rabbit), anti-Fn1 at 1:100 (F3648, Sigma-Aldrich), anti-Aldh1a2 at 1:100 (rabbit, GTX124302, GeneTex), anti-Aldh1a2 at 1:100 (mouse, sc-393204, Santa Cruz), anti-mCherry at 1:500 (chicken, CPCA-mCherry, Encor), anti-mCherry at 1:100 (mouse, 632543, Living Colors), anti-mCherry at 1:200 (rat, M11217, Thermo Fisher Scientific), anti-MLCK at 1:100 (mouse, M7905, Sigma-Aldrich), anti–zf-Cdh5 at 1:100 (rabbit, AS-55715, AnaSpec), p-Smad3 at 1:100 (rabbit, ab52903, Abcam), and anti-Zns5 at 1:100 (mouse, ZIRC 011604). Alexa Fluor–coupled secondary antibodies raised in donkey and goat (Thermo Fisher Scientific) at 1:500 and Phalloidin-Alexa 568–conjugated at 1:200 (A12380, Thermo Fisher Scientific) were used.

For RNA in situ hybridization on paraffin sections, dissected hearts were fixed in sterile 4% PFA at 4°C O/N. Hearts were then washed in 1× diethyl pyrocarbonate (DEPC)–PBS twice for 5 min, followed by 15 to 30 min of incubation through a gradient of ethanol in DEPC-water (50, 70, 80, 95, and 100%) at room temperature. Hearts were then washed in 50% xylene in ethanol and in 100% xylene for 30 min at room temperature, followed by three washes in 100% paraffin at 50°C for 1 hour. Hearts were embedded in paraffin and stored at 4°C and sectioned into 8-μm sections and stored at room temperature. Sections were washed twice in xylene for 10 min each, followed by rehydration in a gradient of ethanol in DEPC-water for 2 min each (100, 95, 80, 70, and 50%). Slides were then washed twice for 5 min with TBST [50 mM tris (pH 7.4), 150 mM NaCl, and 0.05% Tween 20]. Slides were then incubated for 20 min in sterile 4% PFA, followed by two washes in TBST. Slides were then incubated in Proteinase K (0.5 mg/ml) diluted in TBS [50 mM tris (pH 7.4), 150 mM NaCl, and 2 mM CaCl_2_] for 15 min at 37°C, followed by a 5-min wash in ice-cold tris/glycine [50 mM tris (pH 7.4) and 50 mM glycine] to stop the reaction. Slides were then washed twice in TBST, refixed in sterile 4% PFA for 5 min, and washed with TBST. Slides were then immersed in triethanolamine (0.1 M, pH 8.0), and acetic anhydride was added to reach 0.25% under agitation for 12 min. This step is followed by 2× TBST washes, followed by prehybridization in hybridization buffer [50% formamide, 5× SSC, 0.1% Tween 20, heparin (50 μg/ml), yeast t-RNA (500 μg/ml), and 460 μl of 1 M citric acid] at 60° to 65°C for at least 1 hour. Probe (1 μg/ml in hybridization buffer) is denatured at 60° to 65°C for 15 min. Probe is then applied to sections at 60° to 65°C O/N. Slides were then washed in 50% formamide in 2× SSC for 30 min at 60° to 65°C. Slides were then washed at 60° to 65°C for 15 min once with 2× SSC and twice with 0.1× SSC, followed by TBST at room temperature. Slides were then washed at 37°C for 15 min once with 2× SSC and twice with 1× SSC, followed by TBST at room temperature. Slides were then incubated in blocking solution [TBST + 0.5% bovine serum albumin (BSA)] for at least 1 hour at room temperature. Alkaline phosphatase–tagged anti-digoxigenin antibody (1:1000 in blocking solution; Roche) was applied to slides at room temperature for at least 2 hours. Slides were then washed five times with TBST. Prefiltered BM-Purple (Roche) was then applied, and the slides were incubated in a dark, humid chamber until the signal was observed. Slides were then washed with TBST, fixed in 4% PFA for 5 min, and mounted for imaging.

In situ hybridization on wholemount adult caudal fins was performed as described (*33*). Digoxigenin-labeled antisense probes were synthesized using T7 polymerase (Roche) and DIG RNA labeling kit (Roche). The sequences of primers used to amplify probe templates are listed in table S5. Stained samples were imaged on a Nikon SMZ25 stereomicroscope.

### Alizarin Red S staining

Adult zebrafish were sacrificed and fixed O/N in 4% PFA at 4°C on a nutator. After washing with 1× PBS, the fish were stained with Alizarin Red S (0.01% final concentration in 1× PBS) for 1 hour on a nutator, followed by 3× washes with 1× PBS. Imaging was performed using an inverted Zeiss Cell Observer SD confocal microscope.

### Tissue dissociation and cell sorting

Adult zebrafish cardiac endothelial [*Tg*(*fli1:*EGFP)*^+^*] and nonendothelial cells [*Tg*(*fli1:*EGFP)*^−^*] were isolated from a pool of two ventricles per replicate, following the manufacturer’s instructions (Pierce Primary Cardiomyocyte Isolation Kit, Thermo Fisher Scientific), with the following modifications: Incubation was performed at 30°C with gentle shaking for 30 min, followed by careful resuspension in 1× Hanks’ balanced salt solution (Gibco) with 0.25% BSA. Suspended cells were immediately sorted using a FACSAria III (BD) sorter for EGFP^+^ and EGFP^−^ cells. Dead cells were gated out using DAPI staining.

### Gene expression profiling

For the microarray, total RNA was isolated from control versus 96-hpci ventricles and control versus exercised ventricles using the TRIzol-chloroform method. Dual-color cDNA labeling and hybridization were performed by MOgene (commercial service) using the Agilent Zebrafish (V3) 4 × 44 K platform.

For RNA-seq, RNA was isolated from adult zebrafish ventricles and caudal fin tissues using the miRNeasy micro Kit (Qiagen) combined with on-column deoxyribonuclease (DNase) digestion (RNase-free DNase set, Qiagen) to avoid contamination by genomic DNA. RNA and library preparation integrity were verified with LabChip Gx Touch 24 (PerkinElmer). Total RNA (200 to 500 ng) was used as input for TruSeq Stranded mRNA Library preparation following the low-sample protocol (Illumina). Sequencing was performed on the NextSeq500 instrument (Illumina) using v2 chemistry, resulting in minimum of 15 million reads per library with 1 × 75–base pair single-end setup. The resulting raw reads were aligned versus Ensembl zebrafish genome version danRer11 (GRCz11), and the downstream analysis was performed as described (*80*). Cutoffs for identifying differentially expressed genes are as mentioned wherever needed.

### Transcriptomic data reanalysis

We obtained the processed counts per million of the bulk RNA-seq experiment from GEO-GSE95755 (*49*) and the microarray raw data from GEO-GSE111059 (*3*) to calculate FCs and *P* values. We obtained raw count matrices of single-cell RNA-seq from adult zebrafish hearts-GSE106121 (*50*) and regenerating adult zebrafish caudal fins-GSE137971 (*51*) and reanalyzed the data as follows: We calculated the number of expressed genes, total reads, and the percentage of counts assigned to mitochondrial transcripts per cell and filtered low-quality cells with a mitochondrial content exceeding 30%. Next, we filtered genes that were expressed in less than 100 remaining cells. We normalized read counts to the number of total counts using Scanpy, transformed gene expression data into log space, and applied principal components analysis, retaining the top 50 components. Next, we used BBKNN to calculate a batch-balanced k-nearest neighborhood graph using the animal ID as covariate. Uniform Manifold Approximation and Projection (UMAP) was used to embed cells into a two-dimensional space. Further, we used the Leiden algorithm to cluster cells using a resolution of 0.3 to 0.4. Last, the data were visualized using the cellxgene platform.

### Gene ontology analysis

Ingenuity Pathway Analysis (Qiagen) was run with default settings with 180 co-regulated genes (table S1) as a query dataset. For all GSE analyses, R package fgsea was used, by converting zebrafish and mouse gene symbols to human gene symbols. Preannotated gene sets from Hallmark, Kyoto Encyclopedia of Genes and Genomes (KEGG), and Reactome databases were downloaded from Molecular Signatures database (MSigDB, www.gsea-msigdb.org/gsea/msigdb/). All the data, including individual gene names for each gene ontology term, are available in table S4.

### Primary human endothelial culture

HUVECs (Lonza) were cultured in endothelial growth medium (EGM-2, Lonza) using collagen I–coated six-well plates, and only cells of passages *P* < 5 were used. For KD experiments, HUVECs were double-transfected at consecutive days each with 29 nM siRNA (SASI_Hs01_00156548, Sigma-Aldrich) using Lipofectamine RNAiMAX (Invitrogen) and/or treated with Activin type 1 receptor inhibitor (10 ng/ml; SB431542, Calbiochem). Alternatively, cells were stimulated with rhIL-11 (10 ng/ml; CYT-214, Pepnet) and/or rhTGF-β2 (10 ng/ml; 100-35B, Peprotech) in EGM-2 for 96 hours with renewal of culture medium and cytokines every 24 hours.

### Reverse transcription quantitative polymerase chain reaction

For whole ventricles, total RNA was isolated from uninjured and cryoinjured whole ventricles or dissected injured areas using the TRIzol-chloroform method. Single whole-ventricle or injured area per biological replicate, and at least 250 ng of total RNA was reverse-transcribed. For sorted cells, total RNA was isolated using the TRIzol-chloroform method. At least 80 ng of total RNA was reverse-transcribed. For HUVECs, total RNA was isolated using the RNA Clean and Concentrator kit (Zymo Research). At least 500 ng of total RNA was reverse-transcribed. For larval fin fold regeneration, total RNA was isolated from a pool of 20 dissected larvae using the RNA Clean and Concentrator kit (Zymo Research). At least 500 ng of total RNA was reverse-transcribed. All the RT reactions were performed with the Maxima First Strand cDNA Synthesis Kit (Thermo Fisher Scientific) following the manufacturer’s protocol. mRNA levels were normalized against the mRNA levels of *rpl13a* (zebrafish) and *GAPDH* (HUVECs). All reactions were performed in at least technical duplicates using the SYBR Green PCR Master Mix (Thermo Fisher Scientific) on CFX Connect Real-Time System (Bio-Rad). Primer sequences and Ct are as shown in table S5.

### Phylogenetic analysis

Phylograms indicating the orthology of *il11a*, *il11b*, and *il11ra* within the IL-6 family of cytokines and receptors across human, mouse, and zebrafish were established on the basis of the respective full-length primary sequence of their encoded proteins. The tool Phylogeny.fr (www.phylogeny.fr) was run with default settings. Details of peptides used are as shown in table S6.

### Quantification and statistical analysis

Cardiac scar was assessed using consecutive sections, and the quantification was performed on the section with the largest scar area. Trabecular CM protrusion was measured on at least two nonconsecutive 50-μm-thick cryosections. CM proliferation and cortical CM protrusion were measured on at least two nonconsecutive 11-μm-thick cryosections. For measuring endothelial invasion on wholemount ventricles, injured area was determined by the corresponding bright-field images. pSmad3^+^ endothelial cells were quantified using the Analyze particles function in Fiji. Zen 3.2 (blue edition) or NIS-Elements BR Analysis 4.30.00 64 bit or Fiji was used for quantifications.

GraphPad Prism 8 was used to determine the *P* values and perform all statistical analyses. Each sample group was tested for Gaussian distribution using the Shapiro-Wilk normality test. If the data were normally distributed, parametric tests were used: two-tailed Student’s *t* test for comparing two samples. If the data were not normally distributed, nonparametric tests were used: Mann-Whitney *U* test for comparing two samples. For HUVEC experiments, paired two-tailed Student’s *t* test was used for comparing two samples, or repeated measures one-way analysis of variance (ANOVA) with Tukey’s multiple corrections test was used for comparing more than two samples. The exact *P* values and the statistical tests performed are indicated in the figures and figure legends, respectively.

## Notes

View/request a protocol for this paper from *Bio-protocol*.
