# Supplementary material for: Interleukin-11 signaling promotes cellular reprogramming and limits fibrotic scarring during tissue regeneration
Source: Sci Adv. 2021 Sep 8;7(37):eabg6497. doi: 10.1126/sciadv.abg6497 (PMC8442930; doi:10.1126/sciadv.abg6497)
Supplement: Supplementary file 1 — Figs. S1 to S13 Legends for tables S1 to S6 [file sciadv.abg6497_sm.pdf]

Science Advances
AAAS

Supplementary Materials for

Interleukin-11 signaling promotes cellular reprogramming and limits fibrotic scarring during tissue regeneration

Srinivas Allanki, Boris Strilic, Lilly Scheinberger, Yeszamin L. Onderwater, Alora Marks, Stefan Günther, Jens Preussner, Khrievono Kikhi, Mario Looso, Didier Y. R. Stainier, Sven Reischauer*

*Corresponding author. Email: s.reischauer@kerckhoff-fgi.de

Published 8 September 2021, *Sci. Adv.* **7**, eabg6497 (2021)
DOI: 10.1126/sciadv.abg6497

The PDF file includes:

Figs. S1 to S13
Legends for tables S1 to S6

Other Supplementary Material for this manuscript includes the following:

Tables S1 to S6

Figure S1. Allanki et al.

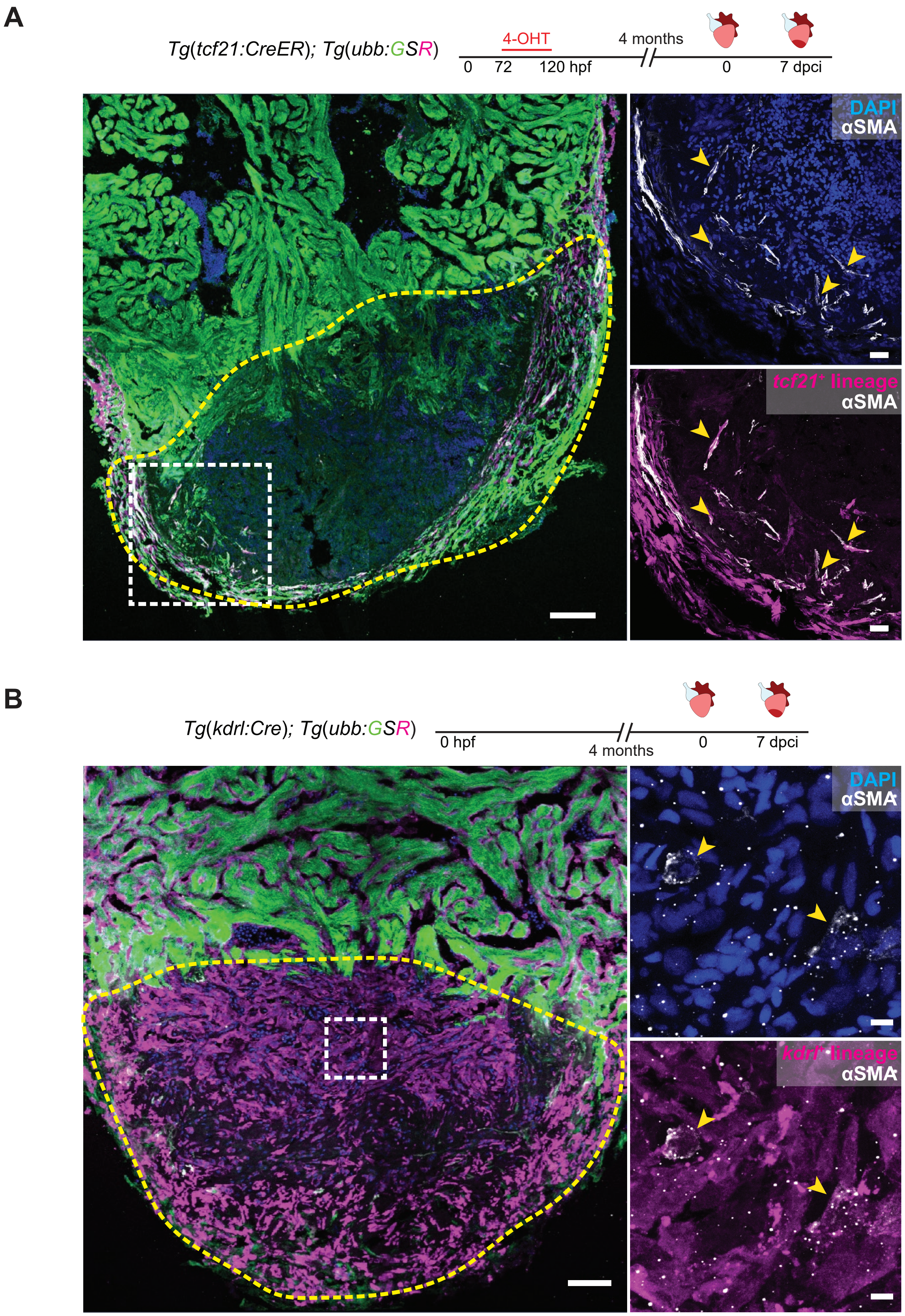

Figure S1. Analysis of myofibroblast origin during zebrafish cardiac regeneration.
(**A**) Experimental design and immunostaining (GFP - green, αSMA – white, and mCherry – magenta; n=4; 7 dpci) on cryosections from *Tg*(*tcf21:CreER*); *Tg*(*ubb:GSR*) ventricles. (**B**) Experimental design and immunostaining (GFP - green, αSMA – white, and mCherry – magenta; n=4; 7 dpci) on cryosections from *Tg*(*kdrl:Cre*); *Tg*(*ubb:GSR*) ventricles. n= ventricles (A, B). Yellow dashed lines demarcate the injured area (A, B); arrowheads point to $αSMA^+$ cells derived from $tcf21^+$ lineage (A insets) and $kdrl^+$ lineage (B insets). Scale bars, 100 µm (A), 50 µm (B), 10 µm (A insets), 5 µm (B insets).

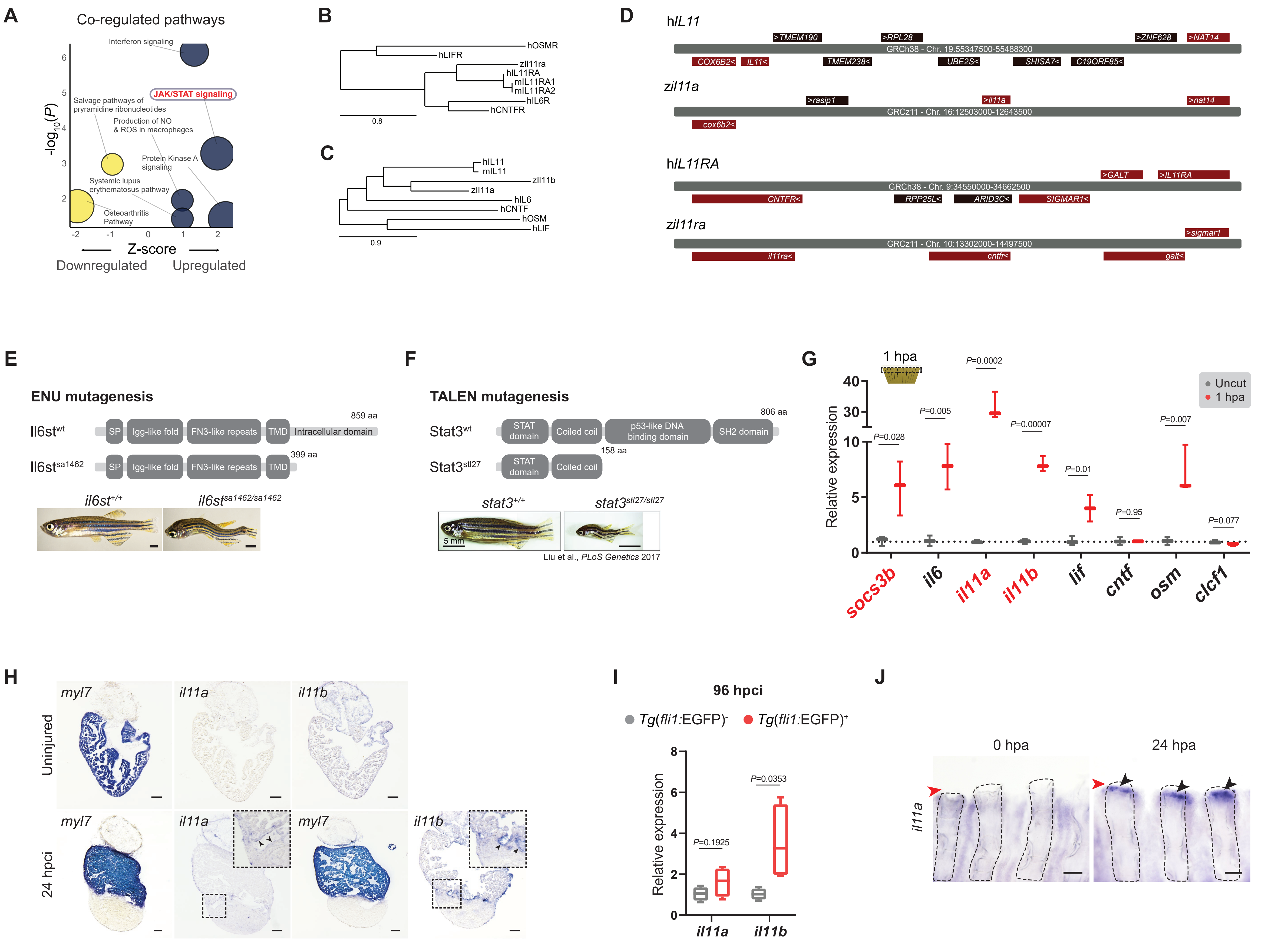
A
Co-regulated pathways
Interferon signaling
JAK/STAT signaling
Salvage pathways of pryramidine ribonucleotides
Production of NO & ROS in macrophages
Protein Kinase A signaling
Systemic lupus erythematosus pathway
Osteoarthritis Pathway
-log10(P)
Z-score
Downregulated
Upregulated
B
hOSMR
hLIFR
zIl11ra
hIL11RA
mIL11RA1
mIL11RA2
hIL6R
hCNTFR
0.8
C
hIL11
mIL11
zIl11b
zIl11a
hIL6
hCNTF
hOSM
hLIF
0.9
D
hIL11
GRCh38 - Chr. 19:55347500-55488300
zil11a
GRCz11 - Chr. 16:12503000-12643500
hIL11RA
GRCh38 - Chr. 9:34550000-34662500
zil11ra
GRCz11 - Chr. 10:13302000-14497500
E
ENU mutagenesis
Il6st wt
Il6st sa1462
SP
Igg-like fold
FN3-like repeats
TMD
Intracellular domain
859 aa
399 aa
il6st +/+
il6st sa1462/sa1462
F
TALEN mutagenesis
Stat3 wt
Stat3 stl27
STAT domain
Coiled coil
p53-like DNA binding domain
SH2 domain
806 aa
158 aa
stat3 +/+
stat3 stl27/stl27
5 mm
Liu et al., PLoS Genetics 2017
G
1 hpa
Uncut
1 hpa
Relative expression
P=0.028
P=0.005
P=0.0002
P=0.00007
P=0.01
P=0.95
P=0.007
P=0.077
socs3b
il6
il11a
il11b
lif
cntf
osm
clcf1
H
Uninjured
24 hpci
myl7
il11a
il11b
I
96 hpci
Tg(fli1:EGFP)-
Tg(fli1:EGFP)+
Relative expression
P=0.1925
P=0.0353
il11a
il11b
J
0 hpa
24 hpa
il11a

Figure S2. Il-11/Il6st/Stat3 pathway is activated post-injury.

(**A**) Results from pathway analysis using Ingenuity pathway analysis (IPA). (**B** and **C**) Phylogenetic analysis of human, mouse, and zebrafish Il-11 cytokine receptor (B) and ligands (C). (**D**) Synteny analysis for human and zebrafish genes encoding Il-11 ligands and receptors; gene sizes are not to scale. (**E** and **F**) Illustration of wild-type and the predicted mutant proteins, gross morphology of adult zebrafish mutants (E, $il6st^{sa1462}$; F, $stat3^{stl27}$) and their respective wild-type siblings. (**G**) RT-qPCR analysis of Il-6 family cytokine mRNA levels (1 hpa, n=3; uncut, n=3) on adult caudal fins. (**H**) Brightfield images of RNA *in situ* hybridization for *myl7*, *il11a*, *il11b* on 11 µm thick cryosections from uninjured and 24 hpci wild-type ventricles. (**I**) RT-qPCR analysis on sorted *Tg*(*fli1:*EGFP)$^{+}$ vs. *Tg*(*fli1:*EGFP)$^{-}$ cardiac ventricular cells (96 hpci, n=4 each). (**J**) Brightfield images of RNA *in situ* hybridization for *il11a* on wholemount adult caudal fins, 24 hpa. SP: signal peptide (E); TMD: transmembrane domain (E). Box plots (G, I) show median, interquartile range (IQR, box margins) and 5th and 95th percentiles (whiskers). Student's *t*-tests (G, I). n= pools of 2 caudal fins (G); n= pools of two ventricles (I). Black arrowheads point to border zone endothelial gene expression (H) and blastemal expression (J); red arrowheads point to the amputation plane (J); black dashed lines outline the fin rays (J). Ct values are listed in table S5. Photo Credit: Adult zebrafish gross morphology images in F were obtained from Liu et al. (*22*). The photos were published under a CC-BY license (hyperlink). Scale bars, 1 mm (E), 5 mm (F), 200 µm (H), 100 µm (J).

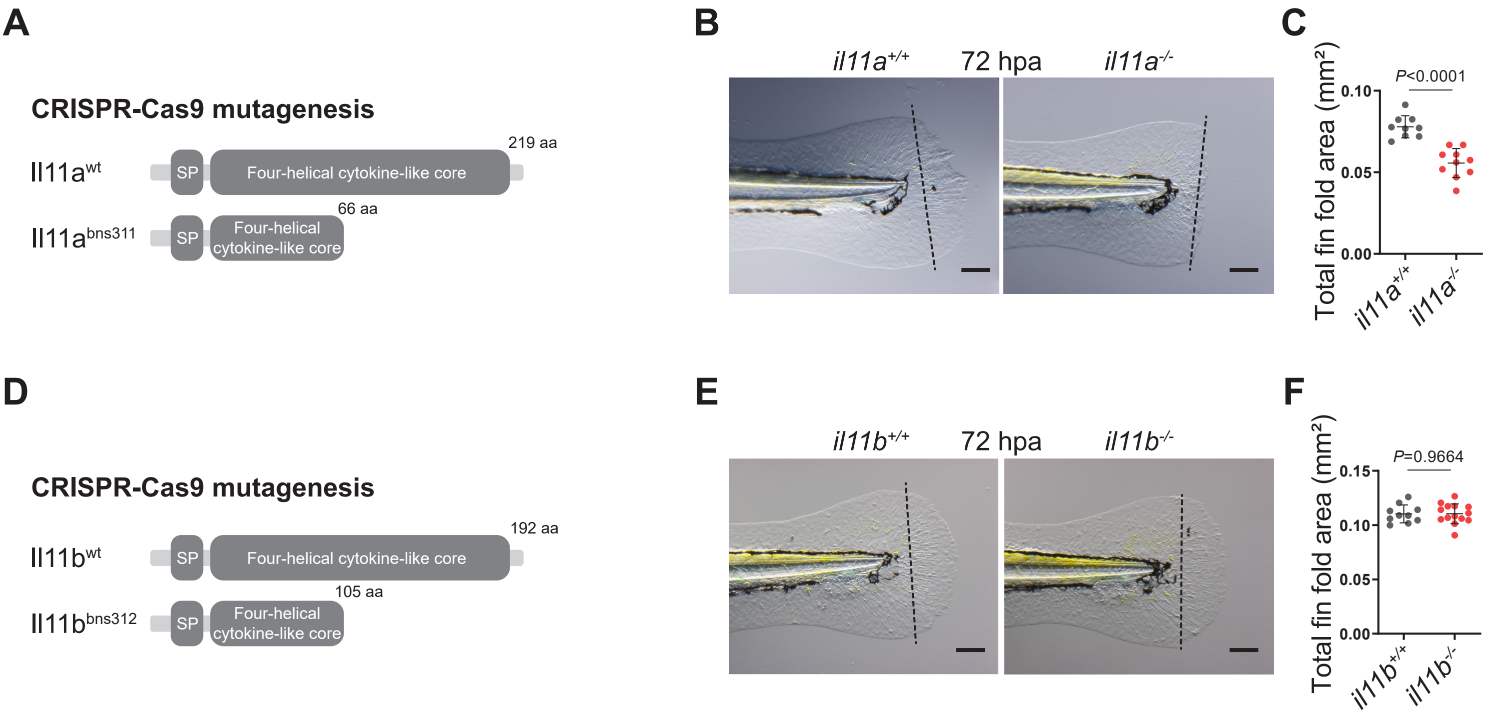
A
CRISPR-Cas9 mutagenesis
219 aa
Il11a^{wt}
SP
Four-helical cytokine-like core
66 aa
Il11a^{bns311}
SP
Four-helical cytokine-like core
B
il11a$^{+/+}$
72 hpa
il11a$^{-/-}$
C
P<0.0001
Total fin fold area (mm^2)
0.10
0.05
0.00
il11a$^{+/+}$
il11a$^{-/-}$
D
CRISPR-Cas9 mutagenesis
192 aa
Il11b^{wt}
SP
Four-helical cytokine-like core
105 aa
Il11b^{bns312}
SP
Four-helical cytokine-like core
E
il11b$^{+/+}$
72 hpa
il11b$^{-/-}$
F
P=0.9664
Total fin fold area (mm^2)
0.15
0.10
0.05
0.00
il11b$^{+/+}$
il11b$^{-/-}$

Figure S3. Il-11a signaling is essential for larval fin fold regeneration.

(**A** to **F**) Illustration of wild-type and the predicted mutant proteins (A, $Il11a^{bns311}$; D, $Il11b^{bns312}$), brightfield images of larval fin fold regeneration (amputated at 48-72 hpf), and their corresponding quantification of the fin fold area at 72 hpa (B and C, $il11a^{bns311}$, wt siblings, n=9; mut, n=10; E and F, $il11b^{bns312}$, wt siblings, n=10; mut, n=14). SP: signal peptide (A, D). Data represent mean ± S.D. (C, F). Student's *t*-tests (C, F). n= larvae (C, F). Black dashed lines demarcate the amputation plane (B, E). Scale bars, 100 µm (B, E).

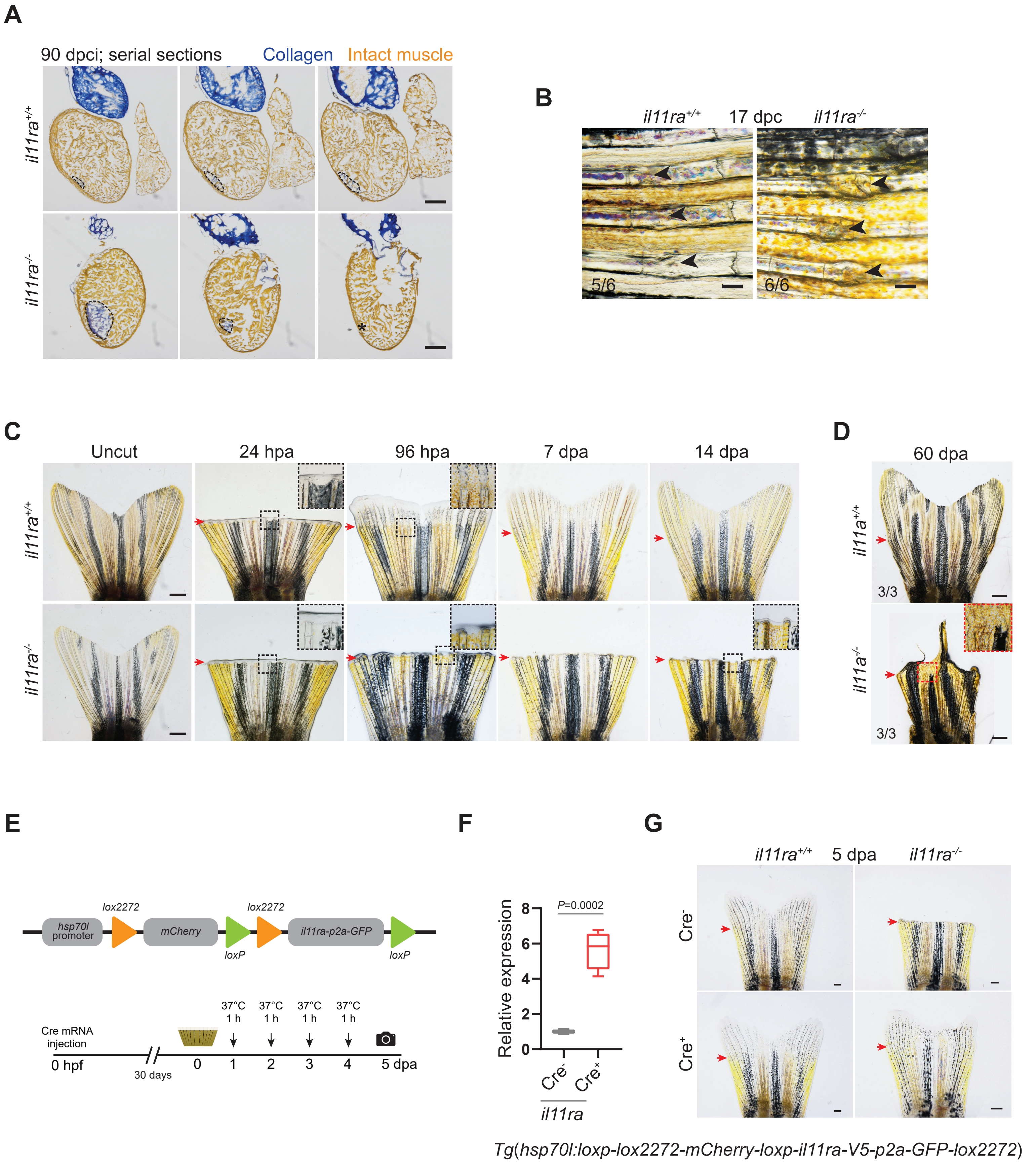
A
90 dpci; serial sections
Collagen
Intact muscle
il11ra+/+
il11ra-/-
B
il11ra+/+
17 dpc
il11ra-/-
5/6
6/6
C
Uncut
24 hpa
96 hpa
7 dpa
14 dpa
il11ra+/+
il11ra-/-
D
60 dpa
il11a+/+
il11a-/-
3/3
3/3
E
lox2272
lox2272
hsp70l promoter
mCherry
il11ra-p2a-GFP
loxP
loxP
Cre mRNA injection
0 hpf
30 days
0
37°C 1 h
1
37°C 1 h
2
37°C 1 h
3
37°C 1 h
4
5 dpa
F
P=0.0002
Relative expression
8
6
4
2
0
Cre-
Cre+
il11ra
G
il11ra+/+
5 dpa
il11ra-/-
Cre-
Cre+
Tg(hsp70l:loxp-lox2272-mCherry-loxp-il11ra-V5-p2a-GFP-lox2272)

Figure S4. Il-11 signaling is essential for adult heart and fin generation.

(**A**) AFOG staining on serial cryosections from *il11ra*$^{-/-}$ vs. wild-type sibling ventricles, 90 dpci, corresponding to Fig. 2H. (**B**) Wholemount images of bone crush injury in *il11ra*$^{-/-}$ (n= 6) vs. wild-type (n= 6) caudal fins at 17 days post crush (dpc). (**C**) Wholemount images of time course of caudal fin regeneration in *il11ra*$^{-/-}$ vs. wild-type siblings. (**D**) Wholemount images of caudal fin regeneration in *il11a*$^{-/-}$ (n=3) vs. wild-type siblings (n=3), 60 dpa. (**E**) Illustration of *il11ra* re-expression construct and experimental set-up. (**F**) RT-qPCR analysis to test the induction of *il11ra* expression in caudal fins of *Tg*(*hsp70l:loxp-lox2272-mCherry-loxp-il11ra-V5-p2a-GFP-lox2272*) zebrafish (n=4 each; Cre mRNA injected vs. uninjected), 5 dpa. (**G**) Wholemount images of fin regeneration at 5 dpa in *Tg*(*hsp70l:loxp-lox2272-mCherry-loxp-il11ra-V5-p2a-GFP-lox2272*) zebrafish in *il11ra*$^{-/-}$ vs. wild-type backgrounds, and with or without Cre mRNA injection at the one-cell stage. Box plot (F) shows median, interquartile range (IQR, box margins) and 5th and 95th percentiles (whiskers). Student's *t*-test (F). n= adult caudal fins (B, D, F). Black dashed lines and asterisk demarcate the injured area (A); black arrowheads point to crushed regions in the adult caudal fins (B); red arrows point to the amputation plane (C, D, G). Ct values are listed in table S5. Scale bars, 200 µm (A, G), 100 µm (B), 1 mm (C, D).

Figure S5. Allanki et al.

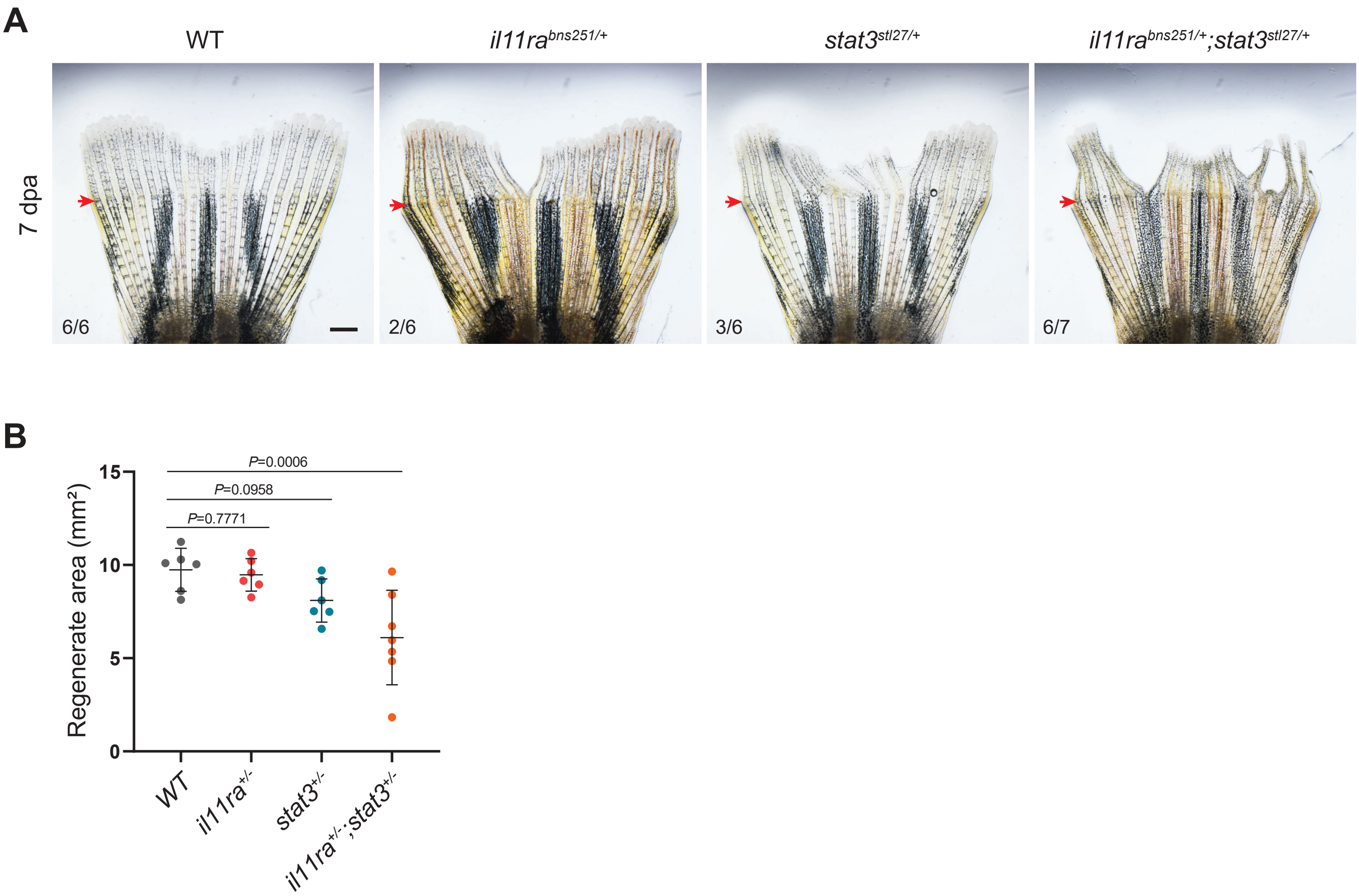

Figure S5. Adult caudal fin regeneration in *il11ra$^{bns251/+}$;stat3$^{stl27/+}$*.

(**A** and **B**) Wholemount images of caudal fin regeneration in *il11ra*$^{+/-}$ (n=6), *stat3*$^{+/-}$ (n=6), *il11ra*$^{+/-}$*;stat3*$^{+/-}$ (n=7) vs. wild-type siblings (n=6) (A) and quantification of the regenerate area, 7 dpa (B). n= caudal fins (A). Data represent mean ± S.D. (B). One-way ANOVA (B). Red arrows point to the amputation plane (A). Scale bar: 1 mm (A).

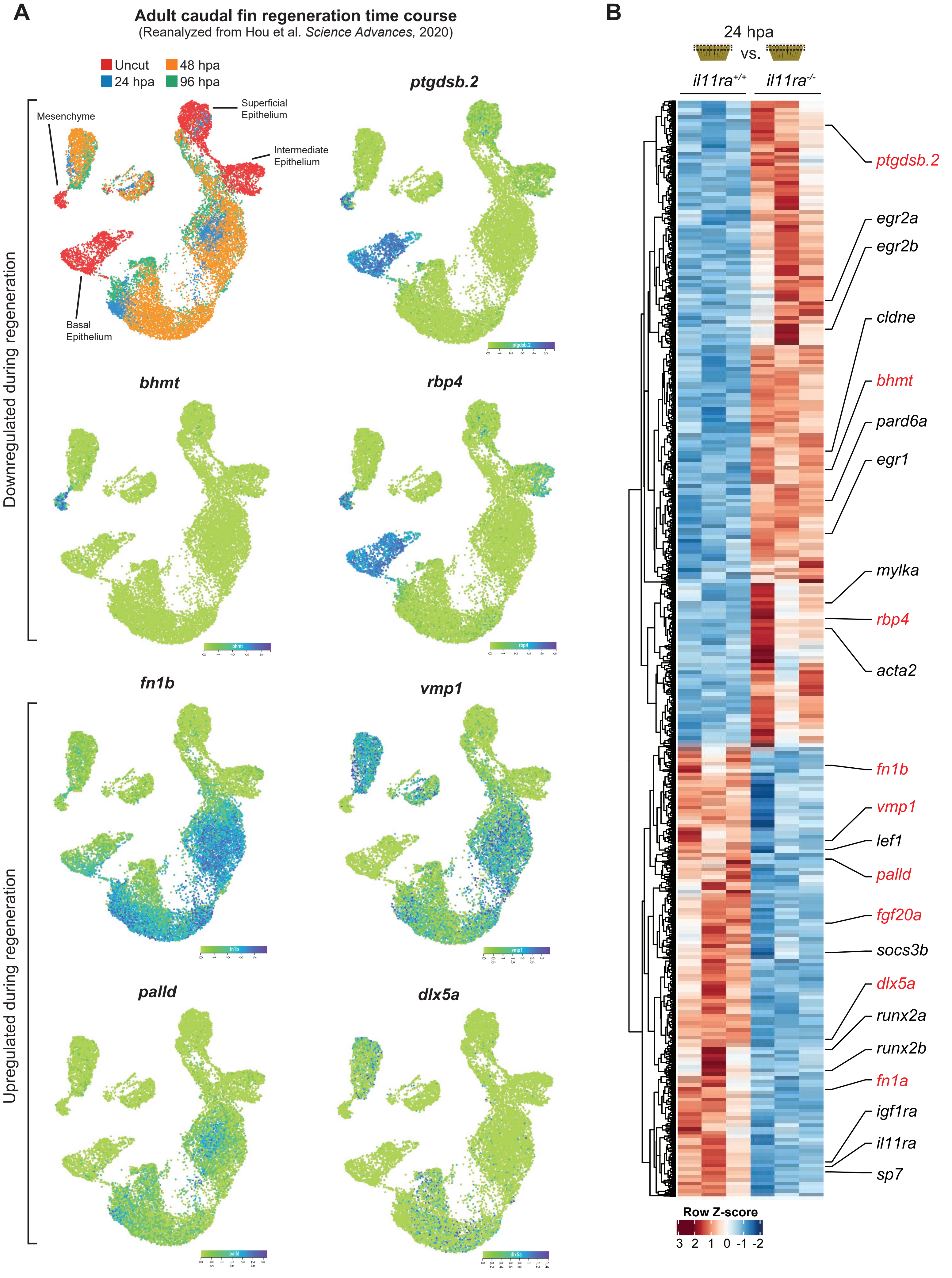
A
Adult caudal fin regeneration time course
(Reanalyzed from Hou et al. Science Advances, 2020)
Uncut
48 hpa
24 hpa
96 hpa
Mesenchyme
Superficial Epithelium
Intermediate Epithelium
Basal Epithelium
Downregulated during regeneration
Upregulated during regeneration
ptgdsb.2
bhmt
rbp4
fn1b
vmp1
palld
dlx5a
B
24 hpa
vs.
il11ra+/+
il11ra-/-
ptgdsb.2
egr2a
egr2b
cldne
bhmt
pard6a
egr1
mylka
rbp4
acta2
fn1b
vmp1
lef1
palld
fgf20a
socs3b
dlx5a
runx2a
runx2b
fn1a
igf1ra
il11ra
sp7
Row Z-score
3 2 1 0 -1 -2

Figure S6. Il-11 signaling is required for reprogramming of regeneration gene expression in the adult caudal fin after amputation.

(**A**) Expression patterns of selected genes from single-cell RNA-seq (reanalyzed from Hou et al., *Sci Adv* 2020) that are specifically upregulated (*fn1b*, *vmp1*, *palld*, *dlx5a*) and downregulated (*rbp4*, *ptgdsb.2*, *bhmt*) during adult caudal fin regeneration that are dysregulated in *il11ra* mutants at 24 hpa. (**B**) Differential expression of zebrafish adult caudal fin regeneration genes in *il11ra*$^{-/-}$ vs. wild-type sibling adult caudal fin transcriptomic analysis, 24 hpa.

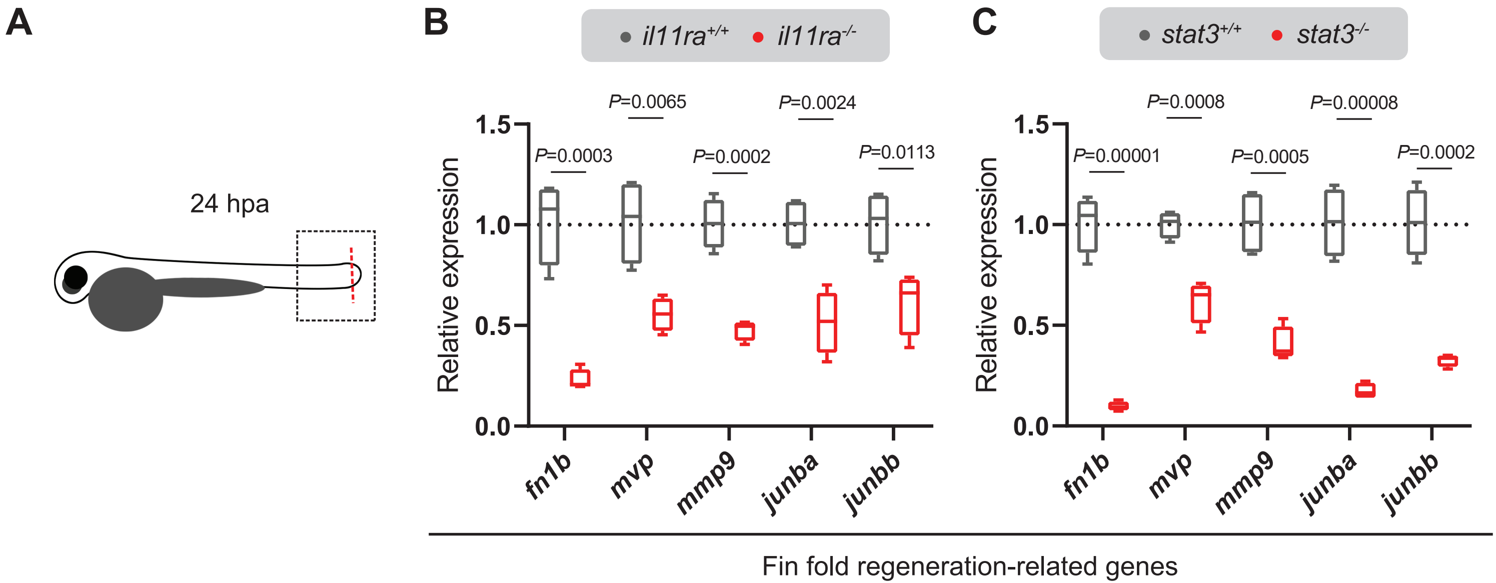
A
24 hpa
B
il11ra+/+ il11ra-/-
Relative expression
1.5
1.0
0.5
0.0
P=0.0003
P=0.0065
P=0.0002
P=0.0024
P=0.0113
fn1b
mvp
mmp9
junba
junbb
C
stat3+/+ stat3-/-
P=0.00001
P=0.0008
P=0.0005
P=0.00008
P=0.0002
Fin fold regeneration-related genes

Figure S7. Comparable deficiency in regeneration gene activation between *il11ra* and *stat3* mutants during larval fin fold regeneration.

(**A** to **C**) Schematic (A) and RT-qPCR analysis on *il11ra*^{bns251} (B, wt siblings, n=4; mut, n=4; 24 hpa) and *stat*^{stl27} (C, wt siblings, n=4; mut, n=4; 24 hpa) for selected fin fold regeneration genes (Yoshinari et al., *Dev Biol* 2009). Box plots (B, C) show median, interquartile range (IQR, box margins) and 5th and 95th percentiles (whiskers). Student's *t*-tests (B, C). n= pools of 20 larval tails (B, C). Black dashed box demarcates dissected tissue processed for RT-qPCR (A); red dashed line demarcates amputation plane (A). Ct values are listed in table S5.

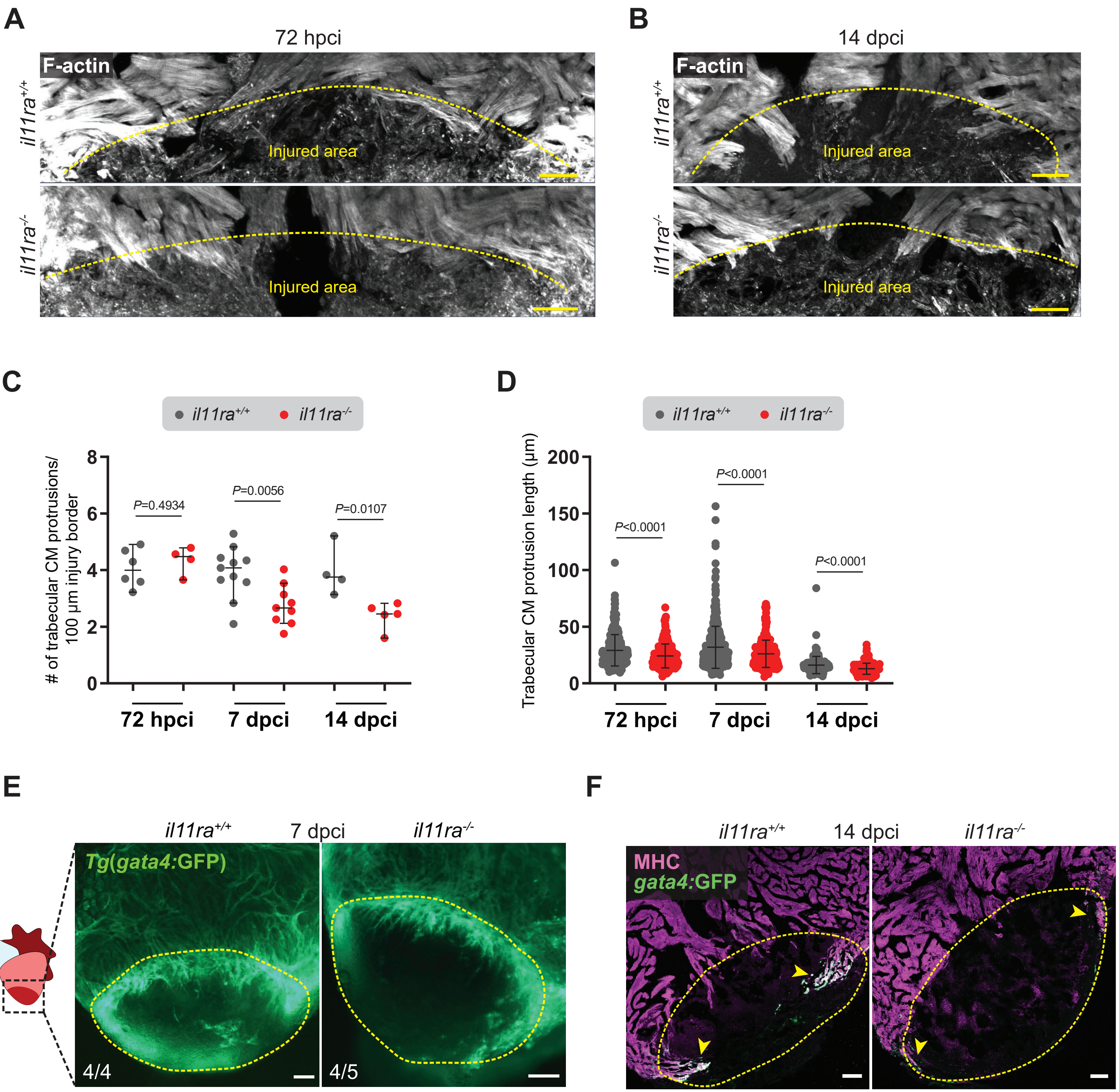
A
72 hpci
F-actin
il11ra+/+
il11ra-/-
Injured area
Injured area
B
14 dpci
F-actin
il11ra+/+
il11ra-/-
Injured area
Injured area
C
il11ra+/+
il11ra-/-
# of trabecular CM protrusions/ 100 µm injury border
P=0.4934
P=0.0056
P=0.0107
72 hpci
7 dpci
14 dpci
D
il11ra+/+
il11ra-/-
Trabecular CM protrusion length (µm)
P<0.0001
P<0.0001
P<0.0001
72 hpci
7 dpci
14 dpci
E
il11ra+/+
7 dpci
il11ra-/-
Tg(gata4:GFP)
4/4
4/5
F
il11ra+/+
14 dpci
il11ra-/-
MHC
gata4:GFP

Figure S8. Defects in cardiomyocyte protrusion in *il11ra* mutants post cardiac cryoinjury. (**A** to **D**) F-actin staining on 50 μm thick cryosections from *il11ra*$^{-/-}$ vs. wild-type sibling ventricles, 72 hpci (A), 14 dpci (B), and quantification of the number per ventricle (C, 72 hpci, wt, n=6; mut, n=4; 7 dpci, wt siblings, n=11; mut, n=9; 14 dpci, wt siblings, n=4; mut, n=5), and length (D, 72 hpci, wt, n=357; mut, n=239; 7 dpci, wt, n=540; mut, n=281; 14 dpci, wt, n=148; mut, n=141) of CM protrusions. 7 dpci data in panels C and D are taken from Fig. 4, D and E, respectively, for comparison. (**E**) Wholemount fluorescence images of *Tg*(*gata4:*EGFP) expression in ventricles (wt siblings, n=4; mut, n=5; 7 dpci). (**F**) Immunostaining (GFP – green, MHC – magenta) on cryosections from *Tg*(*gata4:GFP*) ventricles (wt siblings, n=4; mut, n=5; 14 dpci), corresponding to Fig. 4, F and G. Data represent mean ± S.D. (C) and mean ± S.E.M. (D). Student's *t*-tests (C); Mann-Whitney *U* tests (D). n= ventricles (C); n= CM protrusions (D). Yellow dashed lines demarcate the injured area (A, B, E, F); arrowheads point to protruding *Tg*(*gata4:*GFP)$^{+}$ MHC^{+} cortical CMs (F). Scale bars, 50 μm (A, B, F), 100 μm (E).

Figure S9. Allanki et al.

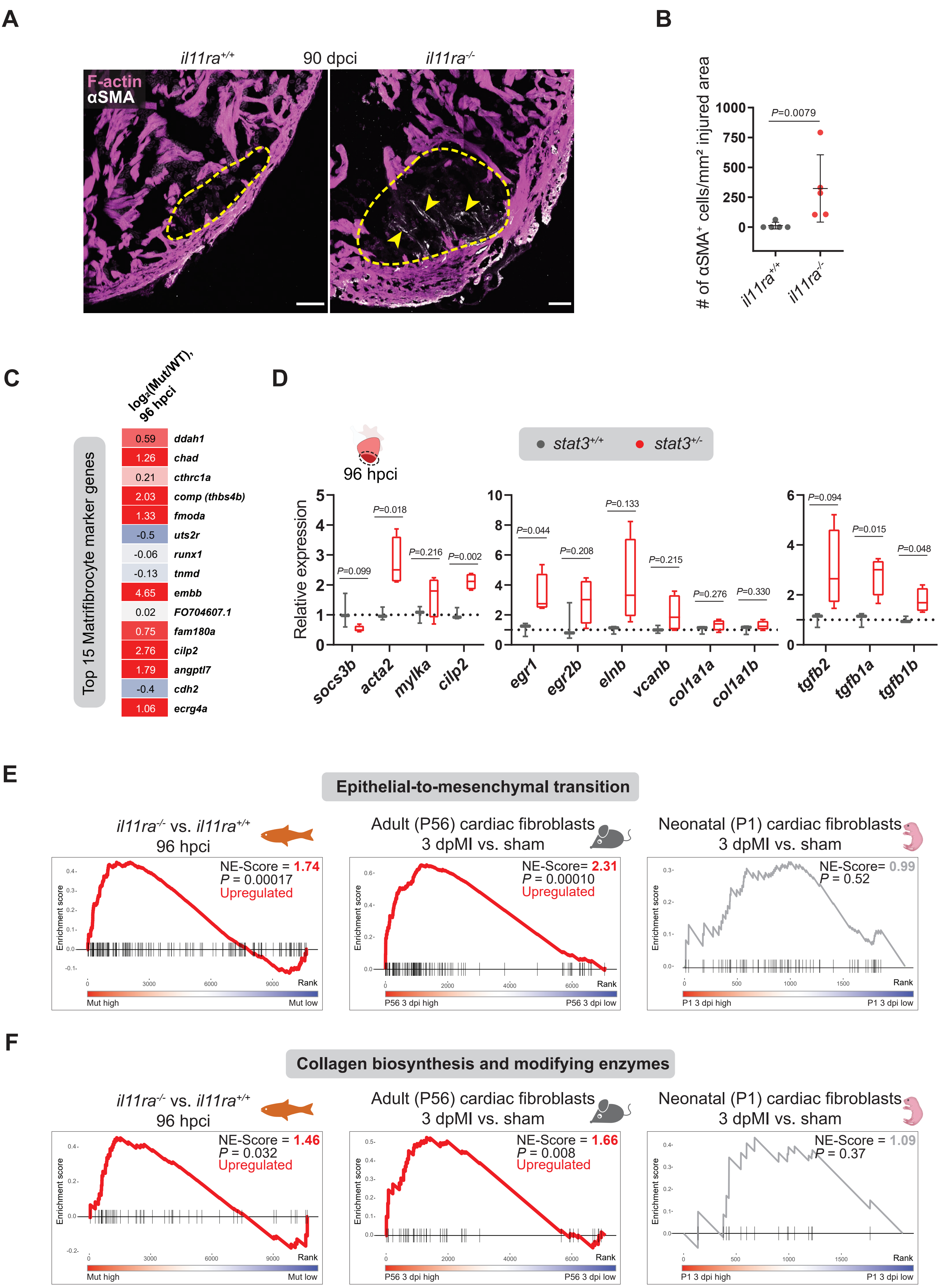

Figure S9. Similarities between non-regenerative *il11ra* mutants and adult mammalian cardiac fibrosis after cardiac injury.

(**A** and **B**) Immunostaining (αSMA – white) and F-actin (magenta) staining on cryosections from ventricles (A, wt siblings, n=5; mut, n=5; 90 dpci) and quantification of αSMA^+ cells in the scar tissue (B). (**C**) Expression of top 15 matrifibrocyte genes (reanalyzed from Fu et al., *J Clin Invest* 2018) in *il11ra*$^{-/-}$ vs. wild-type sibling ventricle transcriptomic profiles, 96 hpci. (**D**) RT-qPCR analysis on dissected injured areas of heterozygous *stat3*^{stl27} (n=4) vs. wild-type sibling (n=3) ventricles for selected fibrosis-associated genes, 96 hpci. (**E** and **F**) GSE analysis plots comparing fibrosis-associated ontology terms between *il11ra*$^{-/-}$ vs. wild-type sibling ventricle transcriptomic profiles (96 hpci) and myocardial infarction (MI) vs. sham from adult (P56) and neonatal (P1) cardiac fibroblast transcriptomic profiles, 3 days post MI (dpMI) (reanalyzed from Quaife-Ryan et al., *Circulation* 2017). Data represent mean ± S.D. (B) and box plots (D) show median, interquartile range (IQR, box margins) and 5th and 95th percentiles (whiskers). Student's *t*-tests (D); Mann-Whitney *U* test (B). n= ventricles (A, D). Yellow dashed lines demarcate the injured area (A); arrowheads point at αSMA^+ cells in the injured area (A). Ct values are listed in table S5. Scale bars, 50 μm (A).

Figure S10. Allanki et al.

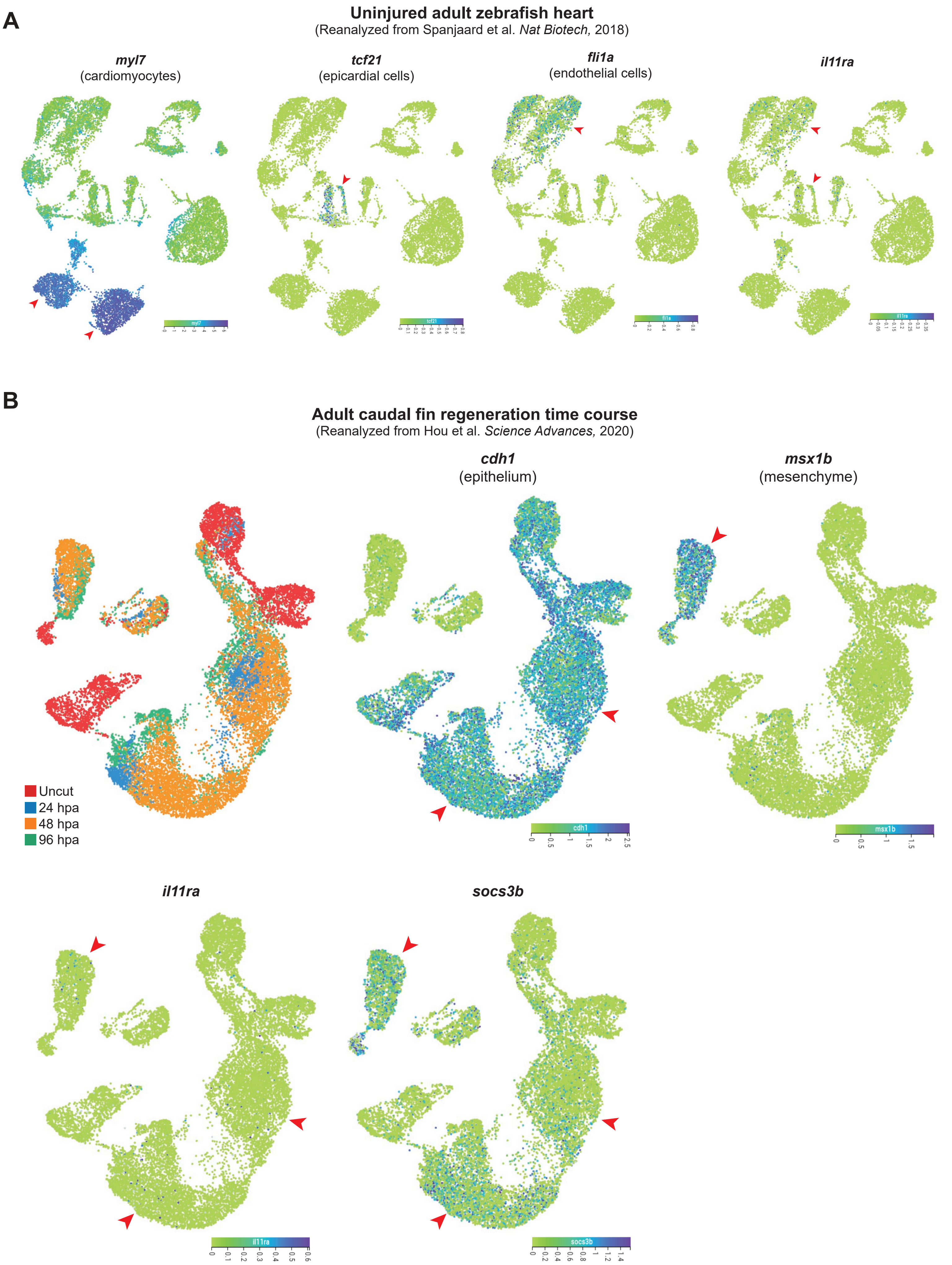

Figure S10. Il-11/Stat3 pathway gene expression patterns at the single-cell level in the uninjured heart and during caudal fin regeneration in adult zebrafish.
(**A**) Expression patterns of selected cell population marker genes and *il11ra* in single-cell RNA-seq data from uninjured adult zebrafish hearts (reanalyzed from Spanjaard et al., *Nat Biotech* 2018). (**B**) Expression patterns of selected cell population marker genes, *il11ra*, and *socs3b* in single-cell RNA-seq data from regenerating adult zebrafish caudal fins (reanalyzed from Hou et al., *Sci Adv* 2020). Red arrowheads point to respective cell clusters (A, B).

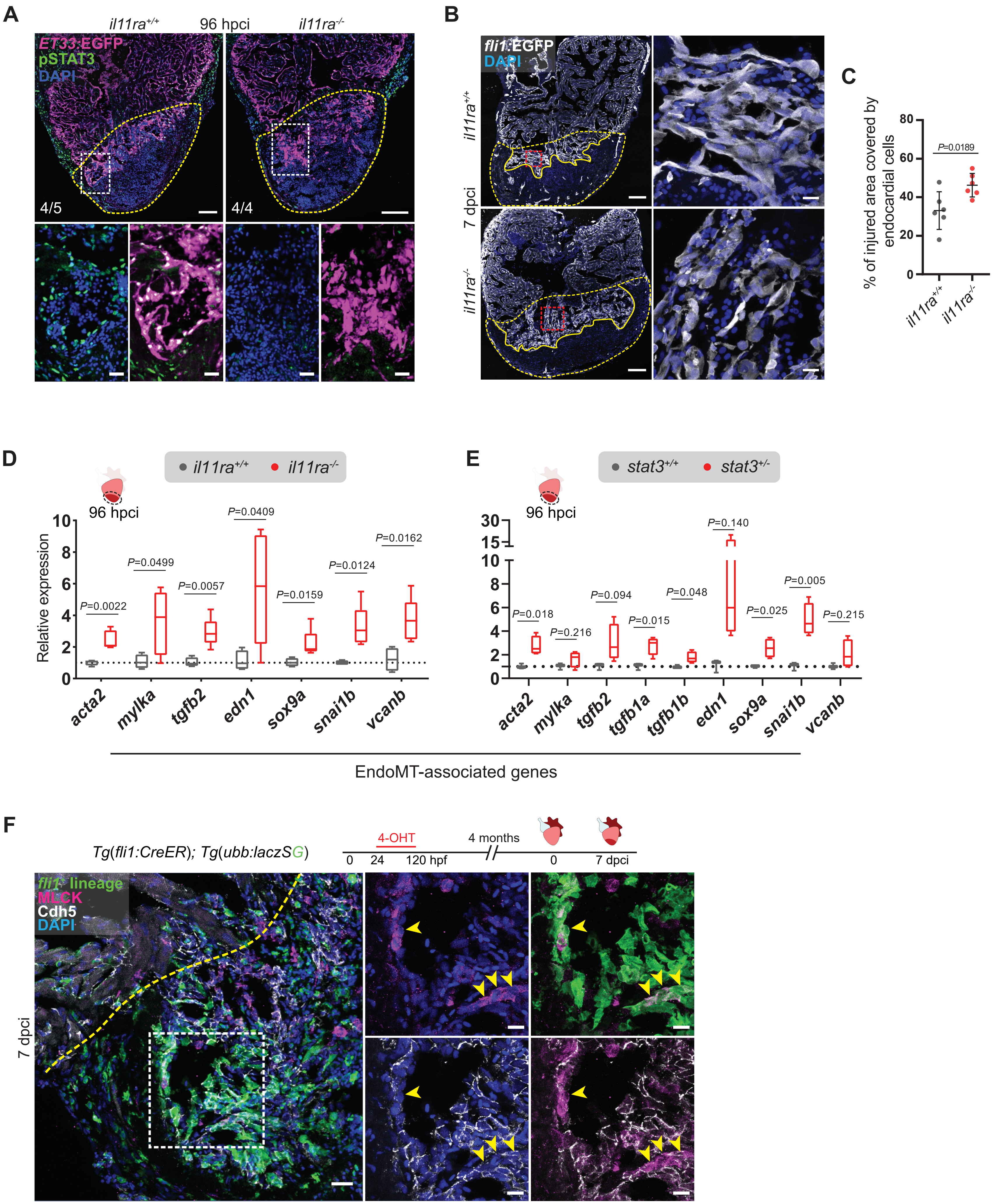
A
il11ra+/+
96 hpci
il11ra-/-
ET33:EGFP
pSTAT3
DAPI
4/5
4/4
B
fli1:EGFP
DAPI
il11ra+/+
7 dpci
il11ra-/-
C
% of injured area covered by endocardial cells
P=0.0189
il11ra+/+
il11ra-/-
D
il11ra+/+
il11ra-/-
96 hpci
Relative expression
P=0.0022
P=0.0499
P=0.0057
P=0.0409
P=0.0159
P=0.0124
P=0.0162
acta2
mylka
tgfb2
edn1
sox9a
snai1b
vcanb
E
stat3+/+
stat3+/-
96 hpci
P=0.018
P=0.216
P=0.094
P=0.015
P=0.048
P=0.140
P=0.025
P=0.005
P=0.215
acta2
mylka
tgfb2
tgfb1a
tgfb1b
edn1
sox9a
snai1b
vcanb
EndoMT-associated genes
F
Tg(fli1:CreER); Tg(ubb:laczSG)
4-OHT
4 months
0
24
120 hpf
0
7 dpci
fli1+ lineage
MLCK
Cdh5
DAPI
7 dpci

Figure S11. Canonical Il-11/Stat3 signaling antagonizes endothelial-to-mesenchymal transition.

(**A**) Immunostaining (GFP – magenta, pStat3 Y705 – green) on cryosections from *Tg*(*ET33:GFP*) *il11ra*$^{-/-}$ (n=4) vs. wild-type (n=5) siblings, 96 hpci. (**B**) Immunostaining (GFP – white) on ventricle cryosections from *Tg*(*fli1:EGFP*) *il11ra*$^{-/-}$ vs. wild-type siblings, 7 dpci. (**C**) Quantification of percentage of injured area covered by *Tg*(*fli1:*EGFP)$^{+}$ endocardial cells (wt siblings, n=6; mut, n=6) from 50 µm thick ventricle cryosections, 7 dpci. (**D** and **E**) RT-qPCR analysis on dissected injured areas of mutant *il11ra*^{bns251} (D, n=5) or heterozygous *stat3*^{stl27} (E, n=4) vs. wild-type siblings (n=4 and 3, respectively) ventricles, 96 hpci, for EndoMT-associated gene expression levels. *acta2*, *mylka*, *tgfb2* and *vcanb* mRNA levels in panel D are taken from Fig. 5E for comparison. (**F**) Experimental design and confocal images of immunostaining (GFP – green, Cdh5 – white, and Myosin light chain kinase, MLCK – magenta) on cryosections from *Tg*(*fli1:CreER*)*; Tg*(*ubb:laczSG*) *il11ra*$^{-/-}$ ventricle, 7 dpci. Data represent mean ± S.D. (C) and box plots (D, E) show median, interquartile range (IQR, box margins) and 5th and 95th percentiles (whiskers). Student's *t*-tests (C, D, E). n= ventricles (A, C, D, E). Yellow dashed lines demarcate the injured area (A, B, F); yellow lines demarcate endocardial invasion in the injured area (B); yellow arrowheads point to *fli1*$^{+}$ derived MLCK^{+} cells (F). Ct values are listed in table S5. Scale bars, 100 µm (A, B), 20 µm (A insets, F), 10 µm (B insets, F insets).

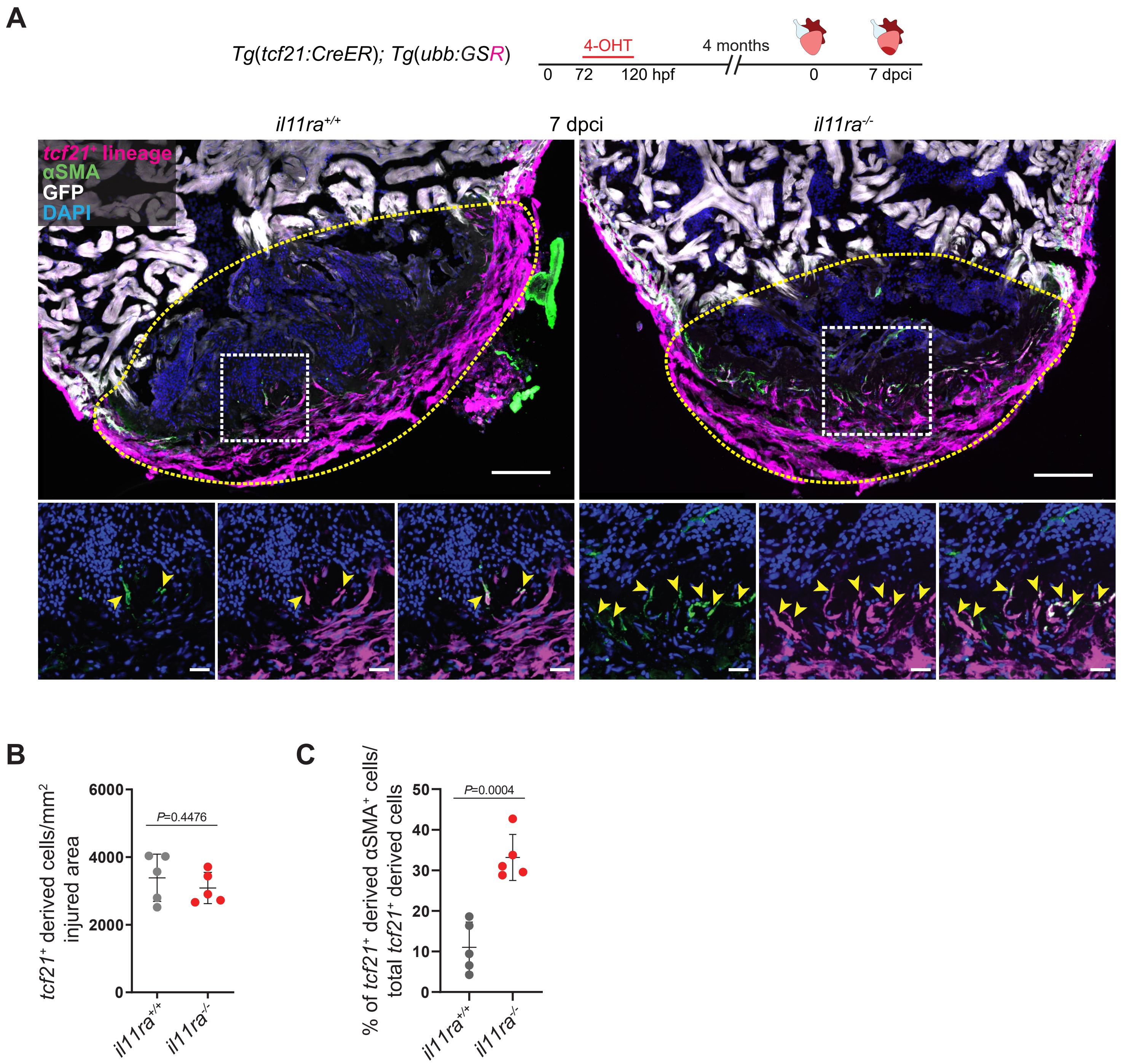
A
Tg(tcf21:CreER); Tg(ubb:GSR)
4-OHT
4 months
0
72
120 hpf
0
7 dpci
il11ra+/+
7 dpci
il11ra-/-
tcf21+ lineage
αSMA
GFP
DAPI
B
tcf21+ derived cells/mm2
injured area
6000
4000
2000
0
P=0.4476
il11ra+/+
il11ra-/-
C
% of tcf21+ derived αSMA+ cells/
total tcf21+ derived cells
50
40
30
20
10
0
P=0.0004
il11ra+/+
il11ra-/-

Figure S12. Il-11 signaling limits epicardial-derived myofibroblast differentiation after cardiac cryoinjury.

(**A**) Experimental design and confocal images of immunostaining (mCherry – magenta, αSMA – green) on cryosections from *Tg*(*tcf21:CreER*)*; Tg*(*ubb:GSR*) ventricles (wt siblings, n=5; mut, n=5; 7 dpci). (**B** and **C**) Quantification of density of *tcf21*$^{+}$ derived cells in the injured area (B) and percentage of *tcf21*$^{+}$ derived αSMA^{+} cells in the injured area (C). Data represent mean ± S.D. (B, C). Student's *t*-tests (B, C). n= ventricles (A). Yellow dashed lines demarcate the injured area (A); yellow arrowheads point to *tcf21*$^{+}$ derived αSMA^{+} cells (A insets). Scale bars, 100 µm (A), 20 µm (A insets).

Figure S13. Allanki et al.

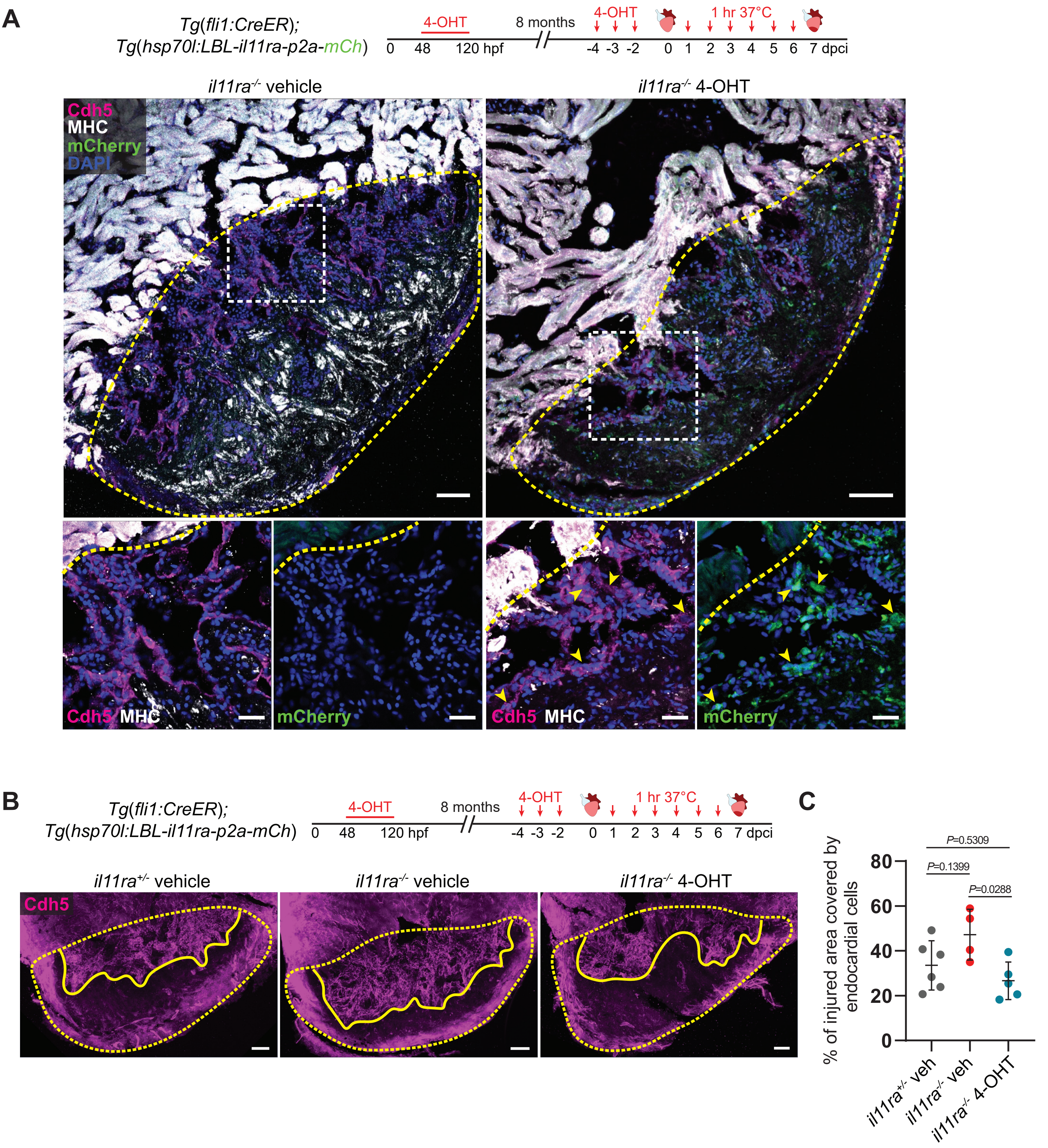

Figure S13. Il-11 signaling in endothelial cells regulates endocardial cell invasion of the injured area after cardiac cryoinjury.

(**A**) Experimental design and confocal images of immunostaining (Cdh5 – magenta, MHC – white, and mCherry – green) on cryosections from vehicle or 4-OHT treated *il11ra*$^{-/-}$ *Tg*(*fli1:CreER*)*;* *Tg*(*hsp70l:LBL-il11ra-p2a-mCh*) sibling ventricles at 7 dpci. (**B**) Experimental design and confocal images of immunostaining for Cdh5 expression on 50 μm thick cryosections from vehicle treated *il11ra*$^{+/-}$ siblings, and vehicle or 4-OHT treated *il11ra*$^{-/-}$ *Tg*(*fli1:CreER*)*;* *Tg*(*hsp70l:LBL-il11ra-p2a-mCh*) ventricles at 7 dpci. (**C**) Quantification of percentage of the injured area covered by Cdh5^{+} endocardial cells (*il11ra*$^{+/-}$ veh, n=6; *il11ra*$^{-/-}$ veh, n=4; *il11ra*$^{-/-}$ 4-OHT, n=5; 7 dpci). Data represent mean ± S.D. (C). One-way ANOVA (C). n= ventricles (C). Yellow dashed lines demarcate the injured area (A, B); yellow lines demarcate the endocardial invasion in the injured area (B). Scale bars, 50 μm (A, B), 20 μm (A insets).

Supplementary Tables

Table S1. Moderate physical exercise vs. cardiac cryoinjury - co-regulated genes in zebrafish ventricles.

Table S2. Canonical pathway and upstream regulator prediction analyses on moderate physical exercise vs. cardiac cryoinjury co-regulated genes.

Table S3. Evolutionarily conserved adult caudal fin regeneration gene expression in *il11ra* mutants vs. wild types at 24 hpa.

Table S4. Gene set enrichment analyses on *il11ra* mutant vs. wild-type ventricles, adult and neonatal mouse cardiac fibroblasts post cardiac injury.

Table S5. Primer sequences and Ct values.

Table S6. Proteins used for phylogenetic analysis.

IL-6 family receptors:

Protein	Encoding transcript ID	Length
hCNTFR	ENST00000351266.8	372 aa
hIL6R	ENST00000368485.8	486 aa

hIL11RA	ENST00000441545.7	422 aa
hLIFR	ENST00000263409.8	1097 aa
hOSMR	ENST00000274276.8	979 aa
zIl11ra	ENSDART00000030976.7	402 aa
mIL11RA1	ENSMUST00000098132.10	432 aa
mIL11RA2	ENSMUST00000179253.1	432 aa

IL-6 family cytokines:

Protein	Encoding transcript ID	Length
hCNTF	ENST00000361987.6	200 aa
hIL6	ENST00000404625.5	212 aa
hIL11	ENST00000264563.7	199 aa
hLIF	ENST00000249075.4	202 aa
hOSM	ENST00000215781.3	252 aa
zIl11a	XM_693882.9	219 aa
zIl11b	ENSDART00000081440.4	192 aa
mIL11	ENSMUST00000094892.11	199 aa
