# Supplementary material for: Interleukin-11 signaling promotes cellular reprogramming and limits fibrotic scarring during tissue regeneration
Source: Sci Adv. 2021 Sep 8;7(37):eabg6497. doi: 10.1126/sciadv.abg6497 (PMC8442930; doi:10.1126/sciadv.abg6497)
Supplement: Supplementary file 2 — Tables S1 to S6 [file sciadv.abg6497_tables_s1_to_s6.zip › sciadv.abg6497_Table_S6.docx]

**Peptides used for phylogenetic analysis:**

IL-6 family receptors

| Protein | Encoding transcript ID | Peptide length |
| --- | --- | --- |
| hCNTFR | ENST00000351266.8 | 372 aa |
| hIL6R | ENST00000368485.8 | 486 aa |
| hIL11RA | ENST00000441545.7 | 422 aa |
| hLIFR | ENST00000263409.8 | 1097 aa |
| hOSMR | ENST00000274276.8 | 979 aa |
| zIl11ra | ENSDART00000030976.7 | 402 aa |
| mIL11RA1 | ENSMUST00000098132.10 | 432 aa |
| mIL11RA2 | ENSMUST00000179253.1 | 432 aa |

IL-6 family cytokines

| Protein | Encoding transcript ID | Peptide length |
| --- | --- | --- |
| hCNTF | ENST00000361987.6 | 200 aa |
| hIL6 | ENST00000404625.5 | 212 aa |
| hIL11 | ENST00000264563.7 | 199 aa |
| hLIF | ENST00000249075.4 | 202 aa |
| hOSM | ENST00000215781.3 | 252 aa |
| zIl11a | XM_693882.9 | 219 aa |
| zIl11b | ENSDART00000081440.4 | 192 aa |
| mIL11 | ENSMUST00000094892.11 | 199 aa |
